# Implications for implementation and adoption of telehealth in developing countries: a systematic review of China’s practices and experiences

**DOI:** 10.1038/s41746-023-00908-6

**Published:** 2023-09-18

**Authors:** Jiancheng Ye, Lu He, Molly Beestrum

**Affiliations:** 1 Weill Cornell Medicine, New York, NY USA; 2 Feinberg School of Medicine, Northwestern University, Chicago, IL USA; 3 Zilber College of Public Health, University of Wisconsin-Milwaukee, Milwaukee, WI USA; 4 Galter Health Sciences Library and Learning Center, Northwestern University, Chicago, IL USA

**Keywords:** Health services, Public health

## Abstract

The rapid advancement of telehealth technologies has the potential to revolutionize healthcare delivery, especially in developing countries and resource-limited settings. Telehealth played a vital role during the COVID-19 pandemic, supporting numerous healthcare services. We conducted a systematic review to gain insights into the characteristics, barriers, and successful experiences in implementing telehealth during the COVID-19 pandemic in China, a representative of the developing countries. We also provide insights for other developing countries that face similar challenges to developing and using telehealth during or after the pandemic. This systematic review was conducted through searching five prominent databases including PubMed/MEDLINE, Embase, Scopus, Cochrane Library, and Web of Science. We included studies clearly defining any use of telehealth services in all aspects of health care during the COVID-19 pandemic in China. We mapped the barriers, successful experiences, and recommendations based on the Consolidated Framework for Implementation Research (CFIR). A total of 32 studies met the inclusion criteria. Successfully implementing and adopting telehealth in China during the pandemic necessitates strategic planning across aspects at society level (increasing public awareness and devising appropriate insurance policies), organizational level (training health care professionals, improving workflows, and decentralizing tasks), and technological level (strategic technological infrastructure development and designing inclusive telehealth systems). WeChat, a widely used social networking platform, was the most common platform used for telehealth services. China’s practices in addressing the barriers may provide implications and evidence for other developing countries or low-and middle- income countries (LMICs) to implement and adopt telehealth systems.

## Introduction

The COVID-19 pandemic introduced extreme burden to health systems globally^1^, highlighting the need for all countries to strengthen the health information technology infrastructure. Telehealth^2^, which employs telecommunications and virtual technologies to deliver care outside of traditional clinical settings, has been proven to be effective in improving healthcare delivery, including patients triage^3^, consultation^4^, treatment^5^, clinical care^6^, and education of healthcare workers and patients^7^.

The pandemic has even more severe impacts on developing countries or low- and middle-income countries (LMICs) due to the limited medical resources and constrained economic situations^8,9^. According to the World Bank, LMICs are defined as economies with a gross national income (GNI) per capita less than $12,535 in 2020^10^. China, the largest developing country, has made significant strides in integrating rural and urban areas, agriculture, and industry into cohesive networks of capital, labor, and commodities in the past decades^10^. Despite these efforts, spatial separation, economic disparities, and differences in social service provision have contributed to persistent rural-urban inequalities^11^. In 2020, the rural population in China was reported to be 38.57% of the total population^12^. Despite the increased investment in healthcare and several major healthcare reforms in China, the rural-urban divide in healthcare resource distribution and health outcomes continues to widen^13–15^. This disparity poses an even larger threat during the COVID-19 pandemic, as timely and high-quality healthcare service is crucial for vulnerable populations in rural areas^16^.

Although previous studies of telehealth implementation in developed countries or high-income countries provide some useful implications, the telehealth systems were often built upon established technological infrastructure with sufficient economic support, well-trained workforces, and target user populations with higher digital literacy^17,18^. These lessons and implications may not be applicable to developing countries or LMICs, where infrastructure is less established, and resources (medical, financial, technological, and human) are limited and unevenly distributed. Therefore, it becomes imperative to understand and share sustainable and scalable strategies for implementing and adopting telehealth systems in developing countries or LMICs. This systematic review aims to comprehensively summarize the characteristics, barriers, and successful experiences in implementing telehealth services in China during the COVID-19 pandemic. This systematic review has three contributions: (1) It offers an in-depth exploration of a specific country, providing detailed insights into the characteristics of telehealth implementation within that particular context; (2) by applying the lens of implementation science, the review synthesizes the findings and offers practical strategies that can be adapted to developing countries or LMICs with varying levels of economic development; (3) providing specific recommendations for building telehealth systems, including guidance on infrastructure and mechanisms, which may be useful to other developing countries or LMICs looking to develop their own telehealth capabilities.

## Methods

This systematic review follows the latest version of the preferred reporting items for systematic reviews and meta-analyses (PRISMA) guideline for identifying potentially related articles^19^. The protocol for this systematic review was available at PROSPERO (CRD42023402844)^20^.

### Data Sources and Search Strategy

A comprehensive search was conducted across five databases: PubMed/MEDLINE, Embase, Scopus, Cochrane Library, and Web of Science, for articles published until the end of June 2022. The search terms were prepared using the PICOS approach, which stands for patients, problem or population (P), issue of interest or intervention (I), comparison, control or comparator (C), outcome (O), and study type (S). To ensure the search’s comprehensiveness and relevance to the research questions, three domains were used to construct the search strategy: COVID-19, telehealth, and China. Studies that were conducted in Hong Kong and Macau were not included because they were developed economies. A combination of keywords and controlled vocabulary terms related to the target concepts was utilized. The search strategy was designed and developed by two authors (JY and LH) independently. To validate the search strategies, an experienced librarian (MB) reviewed and confirmed them. The search strategies for each of the databases were demonstrated in Supplemental Table 1.

### Eligibility criteria

We used the following ***PICOTS*** (**P**opulation, **I**ntervention, **C**omparator, **O**utcomes, **T**ime course, and **S**tudy design) elements to build eligibility criteria for the included studies:

#### *Population*: Any patients

##### Intervention

Studies that evaluated the effect of any intervention, including system- and provider-level implementation strategies as well as patient behaviors, aimed at adopting telehealth services. We included trials that tested interventions targeting:

- Health Systems: health insurance policy; strengthening infrastructure, health system financing, and other incentives (e.g., reimbursement); regulation and governance; and other management techniques.
- Clinicians: education; group problem solving; health care provider-directed financial incentives; supervision; high-intensity or low-intensity training; information and communication technology for health care providers.

- Patients: patient education; insurance and employment improvement; and promotion of self-management (e.g., behavioral and motivational interventions).

##### Comparator

We considered any standard or usual care as the control group.

##### Outcomes

The primary effectiveness outcome was:

- Effectiveness between telehealth and control groups.

Secondary effectiveness outcomes were:

- Cost of intervention.
- Cost-effectiveness.

##### Study design

We included experimental (e.g., randomized methods of assignment) and quasi-experimental (e.g., non-randomized methods of assignment) trials and studies using other experimental designs (e.g., controlled before–after studies or interrupted time series). Exclusion criteria based on study design were observational studies (e.g., prospective/retrospective cohort studies); cross-sectional and case–control studies; and opinion-driven reports (e.g., editorials, letters, and non-systematic or narrative reviews).

### Study selection

The inclusion criteria for this systematic review included (1) studies that utilized telehealth or telemedicine platforms for activities such as screening, triage, prevention, diagnosis, treatment, or follow-up of patients, irrespective of their health conditions, during the COVID-19 pandemic; (2) the review encompassed a broad range of sources, including journals, conferences, scientific guidelines, and case reports, focusing on studies involving human participants with access to the full text (without requiring free article access); (3) studies conducted in either English or Chinese language.

The exclusion criteria for this systematic review included (1) studies that lacked full-text availability; (2) studies that solely assessed participants’ attitudes toward telehealth services without presenting any tangible evidence or results related to the deployment of telehealth platforms or services; (3) studies that used telehealth tools to collect data for testing research hypotheses with no immediate and direct benefits for patients or health care providers; (4) studies that failed to describe any specific telehealth or telemedicine platform, service, or tools used in their research.

### Screening and eliminating irrelevant sources

First, duplicate articles were eliminated from the retrieved articles. Then, JY and LH independently screened articles based on titles and abstracts to identify the studies that potentially could fit into the research question and met the eligibility criteria. An article would be excluded if it was marked irrelevant by two of the reviewers. In cases where the decision couldn’t be made based on the title or abstract alone, the full text was thoroughly examined. In instances of disagreement, the authors held multiple meetings to resolve any discrepancies and achieve a consensus.

### Data extraction

A comprehensive data extraction form was developed to gather general and technical information from the included studies. For each included study, the first author’s name, publication year, research location (city/region), health care system/hospitals, diseases, platforms, outcomes, services, telehealth infrastructure, barriers/challenges, successful experiences, and recommendations were extracted.

Regarding the service type, we considered ten categories, as shown in Table 1. We extracted and categorized challenges and significant barriers from the literature, based on specific topics. The research team members collaborated to reach a consensus, grouping similar topics under relevant categories and assigning appropriate terms to each category based on its theme.

**Table 1:** Purpose of telehealth use during the COVID-19 pandemic.

Purpose	Definition	References	Number of studies
Monitoring	Distance monitoring of patients’ health and/or disease parameters, including clinical data collection, transmission, processing, and management by a health care worker (HCWs) to determine if the patient needs to be in a regular hospital visit or conduct self-management.	^35,49,55,84,85^	5
Consultation	Patients consulted HCWs to assess the condition, answer questions, adjust the treatment plan, or manage complications.	^28,29,33,34,38,51,55,86–91^	13
Triage and making appointment	Using tools by patients or providers to guide them about the necessary actions based on the severity of symptoms, including making an appointment.	^26,28,43,45,55,86,85,91^	8
Diagnosis	Diagnostic support services across geographic distances.	^35,92^	2
Clinical care	Medical or health-related care, including treatment, surgery, physical therapy, etc.	^27,28,30,36,40,52,55,93,94^	9
Follow-up	Making contact with a patient or caregiver at a later, specified date to check on the patient’s progress since their appointment.	^95^	1
Medical education or training	Training HCWs to deliver high-quality, secure, and personalized health care through telehealth; educating patients to receive care through telehealth.	^28,29,72,87,90^	5
Support clinical trial	Identifying eligible patients to participate in clinical trials and providing support.	^31^	1
Knowledge dissemination	Communicating and disseminating COVID-19 and other health knowledge to the public, patients, and caregivers.	^48,55,85^	3
Drug delivery	HCWs use internet-based drug prescription system and provide multi-month dispensing of medications to reduce the need for in-person encounters.	^33,85,86,90,91^	5

### Data synthesis and analysis

The data analysis commenced with an overview of the study and a comprehensive examination of the telehealth system properties, with the extracted data presented in a tabulated format. For articles published in Chinese, the two authors (JY and LH) translated the data into English and engaged in detailed discussions about the information. Simultaneously, the data were systematically categorized to encompass a wide range of values for each variable. We then refined the categories by introducing new ones and consolidating or omitting older versions, persisting until no further new categories emerged, ensuring a robust classification system.

A narrative synthesis was employed to articulate the reported results of the studies. The data obtained were qualitatively elaborated and thoughtfully presented. During the qualitative synthesis, the two reviewers (JY and LH) collaborated closely to identify preliminary themes. Themes that lacked sufficient representation in the data were removed, and similar themes were combined to enhance clarity and cohesiveness^21^.

### Framework

The Consolidated Framework for Implementation Research (CFIR) is a well-established conceptual framework designed to facilitate a comprehensive assessment of multilevel implementation contexts, aiming to identify critical factors that could impact intervention implementation and overall effectiveness^22^. In our study, we employed CFIR as both a theoretical and practical guide to systematically examine various aspects related to the implementation and adoption of telehealth in China. CFIR encompasses five essential domains, namely Outer Setting, Inner Setting, Intervention Characteristics, Characteristics of Individuals, and Process. By leveraging this framework, we meticulously analyzed potential barriers, successful experiences, and recommendations associated with the implementation and adoption of telehealth in China. This approach allowed us to gain valuable insights into the complexities of implementing telehealth services and to develop a robust understanding of the factors influencing its success in the region.

## Results

**Fig. 1:**
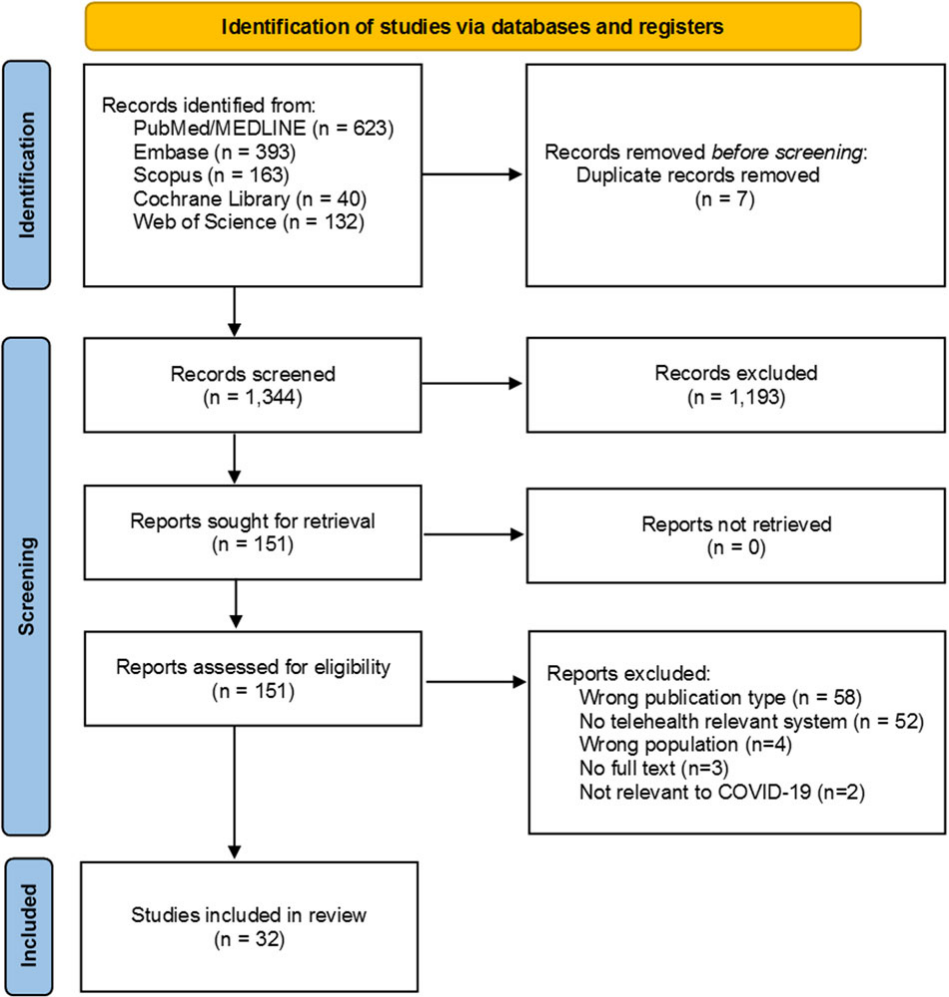
The preferred reporting items for systematic reviews and meta-analyses (PRISMA) flow diagram showing the study selection process regarding the implementation and adoption of telehealth in China.

The screening process is depicted in the PRISMA diagram in Fig. 1. Initially, our search query yielded 1,351 records. To develop and calibrate inclusion criteria, two reviewers (JY and LH) independently screened the titles and abstracts of a randomly selected subset of 100 papers. Subsequently, the reviewers conducted separate screenings, excluding 1,193 papers. The kappa score, measuring agreement between the reviewers, was 0.75. After this initial screening, 151 papers were eligible for full-text review. Finally, 32 papers were included for data extraction. Additional details about the selected papers can be found in Supplemental Table 2.

### Overall statistics

**Fig. 2:**
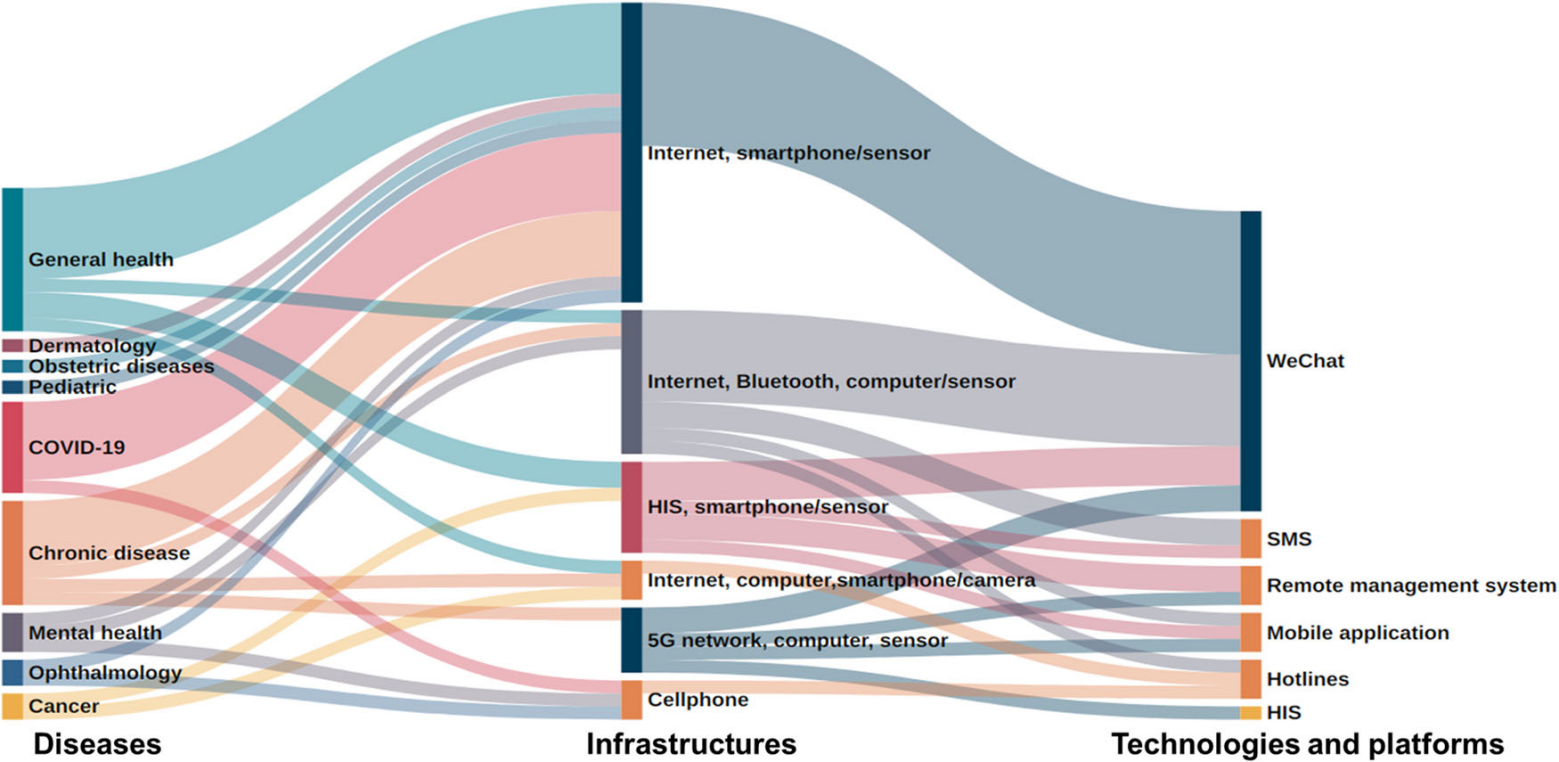
Sankey diagram with diseases, infrastructure, and technologies/platforms of telehealth system in China. HIS hospital information system, SMS Short Message Service.

Of the 32 papers included, most studies (*n* = 28) were published and reported on telehealth services during the early stage of the pandemic (January 2020 to April 2020). The most common service supported by telehealth systems was remote consultation (*n* = 14). Table 2 presents a comprehensive overview of the findings from the included studies. Notably, the majority of the studies (*n* = 21) did not assess or report any clinical outcomes following the implementation of telehealth systems. The most frequently reported outcomes were utilization (*n* = 27), satisfaction (*n* = 11), and effectiveness (*n* = 8) of the telehealth system after implementation. Figure 2 illustrates the distributions among diseases, infrastructure, and technologies/platforms of telehealth systems in China. Most telehealth systems provided services for general health (*n* = 11) and COVID-19 (*n* = 7). Internet and smartphones were identified as the most necessary infrastructure for supporting telehealth services (*n* = 24), with WeChat being the most commonly reported technology in the selected papers (*n* = 18).

**Table 2:** Summary of included telehealth articles (*n* = 32).

Study	Hospital	City	Rural/Urban	Date	Study design	Sample size	Department	Technology/platform	Target disease	Service	Infrastructure	Clinical outcome	Implementation outcome
He^43^	Wuhan Living Room Fangcang Shelter Hospital	Wuhan	Urban	February 2020- March 10, 2020	Observational	1700 patients; 800 healthcare workers	—	HIS	COVID-19	Triage and making appointments	Internet connection	Infection decreased 69.6%	Utilization: Almost 800 doctors, nurses, and lab staff have used
Guo^45^	Wuhan Children’s hospital	Wuhan	Urban	January to October, 2020	Retrospective study	267 outpatient infants	Department of Ophthalmology	WeChat	Retinopathy of prematurity	Triage and making appointment	Internet, smartphone	—	Utilization: 67/86 screening appointments from telemedicine platform.
Fu^31^	Beijing Cancer Hospital	Beijing	Urban	February to April and June to July 2020	Retrospective study	3718 patients	16 departments at Beijing Cancer Hospital	HIS	Cancer	Supporting clinical trials	-HIS	—	Effectiveness: 572 trials; protocol compliance rate of 85.24%; 0 infection rate and error rate. Utilization: 176 clinical research associates;1318 participants in 228 trials
Lian^55^	Guangdong Second Provincial General Hospital	Guangzhou	Urban	December 22, 2019 - April 1 to June 30, 2020	Observational	96,642 patients	—	WeChat	—	Consultation; Screening; Monitoring; Knowledge dissemination	Internet, smartphone	—	Utilization: 96,642 users
Wei^48^	Fangzhuang community health service center	Beijing	Rural	January 23, 2020 - January 29, 2020	Quasi- experimental study	12338 patients and 400 in evaluation	—	An intelligent voice call system	COVID-19	Knowledge dissemination	Call SMS Cellphone	—	Effectiveness: Patients’ knowledge score increased. Utilization: 98,487 voice calls connected, 141,201 messages sent, read rate of 97.8%; Satisfaction:90.8%
Lu^36^	Multi-center	Multiple	—	January 23, 2020 - April 30, 2020	Observational study	16 patients	Department of Neurosurgery	A remote programming system	Chronic intractable pain	Clinical care	Internet, Computer, Sensor	Improvement was achieved in 12 of 13 (92.3%) cases	Utilization:34 sessions with 16 patients; satisfaction: 11 of the 16 (68.8%) patients satisfied
Chen^86^	Multiple hospitals	Multiple	Rural and Urban	February 2020 to 23 February 2020	Descriptive, cross-sectional study	2,599 patients; Over 800 specialists in 347 hospitals	Department of Obstetrics	Mobile application	Obstetric care	Consultation; Monitoring; Drug delivery	Internet, smartphone	—	Utilization: participants used the service for the first time; Satisfaction: 94.63% of the respondents
Zhai^30^	The Children’s Hospital of Fudan University	Shanghai	Urban	February 8 to March 31, 2020	Cross-sectional study	266 experts from 25 pediatric specialties	Outpatient and emergency departments	WeChat	Pediatric disease	Pediatric medical services	Internet, mobile phone	Multiple	The response rate for online consultations was 100%. Utilization: Online visits accounted for 14.7% of all visits. 266 experts from 25 pediatric specialties completed 12,318 effective consultations
Liu^72^	Fujian Provincial Maternity and Child Health Hospital	Fuzhou	Urban	January 2020 to June 2020	quasi-experimental trial	125 mothers with preschool children with autism	—	WeChat	Autism spectrum disorder	Monitor; Consultation	Internet, mobile phone	Anxiety, Depression, Parenting Stress, Hope	Utilization: 40.0% logged their progress in home training each week and 61.5% logged their progress more than 80% of the time for all 20 weeks.; satisfaction: 90.4%
Zhang^87^	Fujian Provincial Maternity and Child Health Hospital	Fuzhou	Urban	December 2019 to May 2020	Retrospective study	190 infants and their parents	Cardiac surgery department	WeChat	Congenital heart disease	Consultation	Internet, mobile phone	Infants’ physical condition; parents’ depression and anxiety	Utilization: all participants
Zhang^92^	Institutions from the STEP	Multiple	Both	October 21, 2019 - March 21, 2020	Longitudinal cohort study	7394 patients	—	A smartphone-based app, WeChat	Hypertension	Home blood pressure measurement	Internet, smartphone/sensor	Systolic blood pressure	Utilization: Wuhan patients more likely to check their BP via the app
Xu^84^	Tongji Hospital of Tongji Medical College	Wuhan	Urban	January 6 - January 31, 2020	Retrospective study	188 individuals	—	WeChat	COVID-19	Monitor	Internet, smartphone	Multiple	Utilization: all participants
Li^95^	Peking Union Medical College Hospital	Beijing	Urban	February 1 to April 30, 2020	Observational study	82 preterm infants	Department of Pediatrics	Telephone and WeChat	Multiple	Follow-up service after discharge	Internet, Smartphone	Prognosis of preterm infants; anxiety level of families	Satisfaction: 96.8% satisfied with online follow-up and 95.2% of parents thought that online follow-up had answered all their questions.
Nan^88^	Beijing Tiantan Hospital	Beijing	Urban	August 2019 to March 2020	Observational study	243 patients	Department of Cardiology and Macrovascular Disease	Tiantanzhixin app	ST segment elevation myocardial infarction (STEMI)	Consultation	Internet, smartphone	Multiple	Effectiveness: reduced pre- and post-hospital delay times in patients with STEMI
Liu^33^	Henan Provincial People’s Hospital	Zhengzhou	Urban	January 24 to February 17, 2020	Retrospective Cohort Study	4589 patients	Multiple	WeChat		Consultation; Drug delivery	Internet, smartphone	—	Utilization: all participants; Satisfaction: 98.1% of survey respondents
Li^34^	University of Hong Kong- Shenzhen Hospital	Shenzhen	Urban	February 19 to March 16, 2020	Observational study	114 patients	Department of Surgery	WeChat	Vascular disease	Consultation	Internet, Smartphone	—	Effectiveness: Better medical advice; Utilization: all participants; Satisfaction: all participants
Li^89^	Beijing Pharmacists Association	Beijing, Tianjin	Urban	February 28 to April 27, 2020	Observational study	39 individuals	Multiple	WeChat	Multiple	Online free pharmaceutical consultations	Internet, Smartphone	—	Utilization: 39 counseling cases; Effectiveness: All consultations completed within 4 h; the completion rate 100%; Satisfaction: 97.4%
Li^93^	Multiple	Jiangsu, Wuhan, Huangshi	NA	26 April 2020 to 9 December 2020	Parallel group randomized controlled trial	119 patients	—	Remote management system	COVID-19	Clinical care	Internet, smartphone	Multiple	Utilization: all participants; Satisfaction: all participants
Li^28^	West China Hospital	Chengdu	Both	1 February to 1 April 2020	Observational study	6662 individuals	Multiple	Huayitong platform	General health	Triage and making appointments; Clinical care; consultation	Internet, smartphone	—	Utilization: 10557 online consultations; only 8.1% (447 of 5517) of physicians used
Ma^52^	9 sub-regional hotline organizations	North Chinese region	/	January to April 2020	Observational study	3,206 calls	—	Hotlines	Psychological assistance	Clinical care	Cellphones	—	Utilization: number of calls received at the regional level hotline (*n* = 3,206) was 0.021% of the population of the region.
Zhou^38^	Third Affiliated Hospital of Sun Yat-Sen University	Guang Zhou	Urban	October 2019 to March 2020	Observational study	3,473 cases		Mobile application	General health	Consultation	Mobile application, internet connection	—	Utilization: Increased utilization during COVID
Zhang^94^	40 hospitals	/	/	January 2019 to March 2020	Descriptive, observational study	196 patients, 909 telemedicine sessions	—	Remote management system	Movement disorders	Clinical care	Computer, camera, wi-fi, internet connection	—	Utilization: 909 telemedicine sessions; Satisfaction: 89% sessions
Chen^27^	Peking Union Medical College Hospital, Huzhou First People’s Hospital	Beijing and Huzhou	Urban	October 2019 to July 2020	Prospective study	6 patients	—	Remote management system	Diabetic retinopathy	Clinical care; Consultation	5 G network, computer, sensor	Experience of visual acuity	System performance: data upload and download speed, network latency
Li^90^	Shanghai Children’s Hospital	Shanghai	Urban	January 2020 to September 2021	Observational study	—	—	HIS; WeChat	General	Consultation; Drug delivery	HIS; Internet, smartphone/sensor	—	Utilization: 30,000 inquiries to clinical pharmacists
Lee^49^		Nanjing	Urban	February 9 to March 16th 2020	Descriptive, observational study	2,088 users	—	Mobile application	—	Monitor	Internet, Bluetooth, smartphone/sensor	—	Utilization: 2,088 users
Lin^40^	Ruijin Hospital	Beijing	Urban	Jan to June 2020	Case study	4 patients	—	Remote management system	Psychiatric disorders	Clinical Care; Consultation	Wifi, Bluetooth, computer	OCD symptoms, (BDI) and anxiety (BAI)	—
Ding^91^	People’s Hospital of Baoan Shenzhen	Shenzhen	Urban	March 2 to April 20, 2020	Descriptive, observational study	1,380 prescriptions	—	Mobile application	General health	Consultation; Drug delivery	Internet, computer, smartphone/camera	—	Utilization: 1380 patients
Mu^51^	Peking University People’s Hospital	Beijing	Urban	Jan 21 to Apr 4, 2020	Descriptive, observational study	698 patients	—	Mobile application	Dermatology	Consultation	Internet, computer, smartphone/camera	—	Utilization: 698 patients used the system
Shao^85^	Taizhou Public Health Medical Center	Taizhou	Urban	January 23 2020 to March 15 2020	Descriptive	—	Multiple	WeChat, Mobile application, Hotline	COVID-19	Consultation; Knowledge dissemination	Internet, computer, smartphone/camera	—	Utilization: 84,255 consultations through the system; 15,510 follow-ups
Wang^29^	Gansu Provincial Hospital	Gansu	—	January 21 to February 29 2020	Survey, observational study	1,043 remote consultations	Multiple	Mobile application	General health	Consultation	Internet, computer, smartphone/camera	—	Utilization (patients): 1,043 consultations; Efficiency: 76.03% cases less than 2 h; Satisfaction 91.8% for providers, 68.42% patients
Han^26^	Multiple centers	—	—	January 2020	Survey, observational study	64 patients	—	Mobile application	Chronic pain	Clinical care	Internet, Bluetooth, sensor	—	Utilization: 81.3% patients used; Satisfaction: 96.7%; Perceived clinical improvement: 96.7%; System failure: 4.8% of patients
Zhang^35^	Sir Run Run Shaw Hospital	Zhejiang	Urban	February 3rd 2020 to February 9th 2020	Observational study	32 patients	Department of Pulmonary and Critical Care Medicine	Remote management system	COVID-19	Monitor	Internet connection, sensor	—	Efficiency: less time

HIS hospital information system.

We mapped the major barriers and successful experiences identified from the papers to the domains on the CFIR (Table 3).

**Table 3:** Identified barriers, successful experiences, and recommendations for implementation and adoption of telehealth during the COVID-19 pandemic based on CFIR framework.

CFIR domain	Barrier	Successful experiences	Recommendations
Outer setting	• Unequal medical and technological resource distribution^27,87^. • Lack of standardized guidelines and supporting evidence^30,31^.	• Provided care to patients in resource-limited areas^30,91^.	• Decentralization of resources from core hospitals to resource-constrained areas^29^. • Expand commercial insurance to cover telehealth^32^.
Inner setting	• Lack of long-term evaluation methods to establish the clinical value^33,34^. • Insufficient human resources and inadequate incentives^31^.	• Reduced use of medical supplies and human resources^35^.	• Design new workflow and reimbursement policies to accommodate telehealth services^28^.
Intervention characteristics	• Unstable internet quality^36^. • Not friendly to users with low digital literacy^51^. • Different health care services require different modalities (e.g., video, audio)^37^. • Privacy and security concerns^49^. • Low diagnostic accuracy^38^.	• Lowered barriers (easy to use and affordable) for diverse patient populations^87,96^. • Reduced adverse events^40^. • Supported multiple functions and smooth patient-provider communication^45^.	• Use off-the-shelf and already widely accepted technologies^37^. • Provide flexible modalities for different situations^45^. • Develop relevant infrastructure before the pandemic^28^. • Integrate telehealth systems with multiple functions^43^. • Develop inclusive and accessible technologies^49^. • Conduct dedicated outreach and provide technical support to persons with limited access or familiarity with new technologies^28^.
Characteristics of individuals	• Low acceptance and buy-in^48^. • Preference for face-to-face interactions^95^.	• Increased evidence base (especially with rigorously designed studies) to improve access or care at a reasonable cost^96^.	• Support from family caregivers^49^. • Combine remote care with in-person care^31^.
Process	• Lack of public awareness^52^. • Inadequate motivation and training^37^. • Additional workload for providers^37^.	• Increased awareness of telehealth among the public^52^. • The train the trainer model alleviated workload for providers who were in high demand^37^. • Partnered with third-party technology companies^55^.	• Use various social media channels to promote public awareness^52^. • Decentralize tasks to primary care^48^. • Tailoring systems based on the different patient populations or hospitals with different levels of technology infrastructures^29^. • Develop and use interchangeable EHRs to facilitate sharing of information among diverse providers^37^.

### Outer setting

Limited medical resources in rural areas pose a significant challenge in China, exacerbating health inequity in settings with scarce resources. As of 2020, the scarcity of licensed physicians nationwide led to a mere 1.56 village clinicians and assistants per 1000 rural residents on duty^23^. Furthermore, access to quality rural healthcare services is predicted to worsen due to the retirement of current practitioners and the allure of better opportunities for new healthcare workers in urban areas^24^. Due to the unequal distribution of medical resources^15^, patients often have to travel to different places to receive high-quality care^25^. With telehealth systems, patients can obtain such care at home, eliminating the burden and costs associated with traveling^26^. Moreover, these telehealth systems empower high-level hospitals, like tertiary hospitals, with abundant medical resources, to share their expertize and support primary and secondary hospitals that may be overwhelmed with patient load. Despite the benefits, rural areas have lagged in the implementation and adoption of telehealth systems^27^. During the pandemic, efforts were made to bridge this gap by enabling urban and tertiary hospitals to extend their medical expertize and human resources to rural and resource-limited areas^28,29^. Patients could remotely consult specialists at the core hospitals or order medications through online pharmacy systems^29^.

As promising as telehealth systems are, during the pandemic, the lack of standardized guidelines and protocols posed challenges in their seamless integration into clinical settings^30,31^. Immediate responses were required, and the necessary guidelines were yet to be developed. In response, customized policies, such as insurance coverage for telehealth visits, played a crucial role in facilitating the introduction of telehealth systems while alleviating the additional workload burden on healthcare workers and the financial strain on patients^32^.

### Inner setting

Telehealth implementation in healthcare organizations may face challenges in evaluating its long-term impact on patients’ clinical outcomes sustainably^33,34^. Hospitals encounter difficulties in determining how to integrate telehealth systems into their existing clinical practices effectively. Moreover, implementing such systems requires additional human resources for development, maintenance, and operation, which can be particularly burdensome for healthcare organizations that are already facing staff shortages during the pandemic^31^.

To facilitate the successful implementation and adoption of telehealth systems without adding extra burdens to health professionals, some hospitals in China have designed innovative workflows that seamlessly integrate telehealth services into their existing practices without disrupting clinical work. For example, the West China Hospital’s intensive care units (ICU) developed a system enabling less experienced bedside physicians to communicate with a multidisciplinary team of senior and experienced physicians through videoconferencing before the COVID-19 pandemic^28^. This platform was adapted during the pandemic to provide telehealth services while maintaining the existing workflow and avoiding the need for significant additional investment.

To ensure the success of telehealth initiatives, we recommend conducting both short-term and long-term evaluations of the implemented systems. These evaluations can help increase buy-in and support from key stakeholders within the organizations. Additionally, gathering feedback and perceptions from relevant stakeholders can further enhance the effectiveness of telehealth systems. Some hospitals have reported positive outcomes with the introduction of telehealth, such as reducing the use of medical supplies and human resources. For instance, Zhang et al. found that implementing a vital sign telemetry system eliminated the need for nurses to check individual patients’ vital signs in-person, leading to a significant reduction in the consumption of personal protective equipment^35^.

### Intervention characteristics

The technical features of the telehealth systems themselves may also hinder successful implementation and adoption. One concern is the stability and quality of telehealth services, especially synchronous patient-provider videoconferencing, where disruptions can occur, affecting remote consultations^36^. In order to accommodate and expand the coverage of telehealth systems, alternatives such as Short Message Services (SMS) could be utilized, which were easy to use, and affordable among populations with lower economic status, and could be applicable to a broader user group^30^.

Certain medical specialties, such as dermatology, heavily rely on images or video-based telehealth systems to ensure accurate diagnoses. However, the effectiveness of such systems depends on patients having access to devices with high-resolution cameras and a stable internet connection^37^. When telehealth encounters fail to provide adequate information, patients may receive inaccurate diagnoses due to insufficient data available to the providers for clinical decision-making^38^.

Privacy and security concerns also come into play during telehealth sessions, with instances of “Zoombombing” being a notable example. Such incidents can expose sensitive protected health information (PHIs) to unauthorized and malicious parties when commercial third-party software is used for telehealth services^39^. To safeguard patient information, it is imperative to have a dedicated Information Technology (IT) team that assesses the security features of the telehealth system, implements end-to-end encryption, and mandates the use of passwords and meeting locks for enhanced protection^39^.

On the positive side, telehealth systems supporting advanced services, like real-time clinical treatments, have been associated with minimal or no adverse events^40^. These platforms have the capacity to collect and analyze patient data on a large scale, offering valuable insights for population health management^41^. Additionally, hospitals can identify trends, risk factors, and best practices to optimize clinical decision-making^42^. A versatile telehealth system is capable of supporting multiple functions^43^, (e.g., consultation, making appointments, follow-up, medication orders, etc.)^44^ and various modalities (e.g., text, audio, video, etc.), thereby providing comparable services to in-person visits^45–47^.

### Characteristics of individuals

Besides technical challenges, human factors are also known to pose significant barriers to the widespread adoption of telehealth systems. Patients, particularly those with lower digital literacy or limited access to technologies, may exhibit lower acceptance of telehealth systems^48^. The process of training and educating patients to use these systems can place a burden on the telehealth IT team^37^. In such cases, family caregivers have emerged as crucial stakeholders, providing essential support to patients facing technology-related challenges. For instance, for older adults using a home quarantine monitoring system that requires a stable internet connection and wearable device configurations, family caregivers who are more tech-savvy can play a vital role in assisting them^49,50^. Certain patient groups have expressed a preference for face-to-face interactions as it allows them to better communicate their health concerns^48^. To address this, some hospitals have adopted a hybrid approach, combining remote telehealth care with in-person visits, aiming to accommodate patients’ individual needs based on their health concerns and reasons for seeking medical attention^19^.

Moreover, users with lower digital literacy may exhibit reduced motivation to utilize telehealth systems. A prime example is older adults, who have been reported as the smallest user group consulting dermatologists through mobile applications for remote diagnosis^51^. To promote the acceptance of telehealth among such users, hospitals may opt to adopt widely used technologies or platforms like WeChat, rather than developing new and complex technologies^37^. This approach could potentially bridge the digital literacy gap and encourage more users to embrace telehealth services.

### Process

Macro-level factors in society may impact the successful implementation of telehealth systems, necessitating an appropriate dissemination approach to streamline the process. For instance, a lack of awareness among the public about available telehealth services, such as mental health hotlines, may result in underutilization^52,53^. To address the issue, mental health hotline centers have effectively encouraged current users to share their positive experiences with telehealth systems on social media platforms^52,54^.

The adoption of telehealth systems requires comprehensive training for healthcare professionals, which can increase their workload, particularly during a pandemic^37^. Ku et al. found that therapists expressed lower satisfaction rates with a rehabilitation app because they had to invest considerable time in assisting patients with app installation^37^. Furthermore, clinicians may lack adequate training in using telehealth systems. To resolve this, some hospitals have adopted the “train the trainer” model, where trained staff pass on their knowledge of telehealth to new users^37^. This strategy effectively reduced the cost of training clinicians and facilitated their onboarding experiences with telehealth.

The development of telehealth systems during a pandemic may be impeded by a lack of technical and human resources within hospitals to build large-scale, robust systems. To overcome this challenge, partnering with third-party technology companies has been found to greatly accelerate the process and provide more scalable solutions beyond what hospitals could achieve independently^48,55^. Tailoring the telehealth systems based on the available technology infrastructure and the needs of the targeted patient populations is crucial, rather than implementing all components, some of which might not be affordable or accessible^29^.

### Strategic development of telehealth system

China has provided valuable insights into promoting and supporting the development of telehealth systems. Since 2018, the Chinese government has implemented policies to encourage the growth of “Internet + health” initiatives, which involved establishing a “national telehealth network”^56^ In late 2019, an important milestone was achieved when online healthcare services were integrated into the national health insurance reimbursement system, a significant step towards digitizing the entire patient journey^56,57^. Furthermore, China made considerable progress in 2019 by launching one of the world’s largest fifth-generation (5 G) networks, significantly enhancing real-time coordination and capabilities in digital and telehealth services^58^. 5 G in health was critical for the “remote consultation platform” of Fangcang shelter hospital, for example, which connected its physicians with senior experts far away in Beijing in real-time^43^.

For other developing countries to effectively implement and adopt telehealth systems, they should consider creating an enabling environment based on their unique characteristics, paving the way toward a national healthcare ecosystem. While the long-term goal for developing countries may be to build a comprehensive telehealth ecosystem, their immediate priorities for implementation can differ based on their current state. Countries like China, India, Brazil, and Lebanon have already successfully implemented telehealth systems that proved to be extensively useful during the COVID-19 pandemic^59^. However, many LMICs encountered challenges in integrating telehealth systems into their existing healthcare infrastructure. One of the main concerns in LMICs might be the high initial costs associated with technology, as well as the need for reliable internet connectivity and coordination between various sectors and stakeholders, such as health ministries, science and technology ministries, local governments, and different-level hospitals^60^. In LMICs, government approval is typically required for launching telehealth programs, necessitating clear regulations, legislation, and funding to facilitate system development and implementation, which could lead to delays in adopting telehealth, especially during the crisis like the COVID-19 pandemic^61^.

We summarized the various infrastructures and telehealth system in Table 4, which may provide practical telehealth guidelines for developing countries or LMICs that have different and heterogenous infrastructures. We also provided example studies in different developing countries to demonstrate how the framework may be applied to different contexts. The basic and fundamental level of infrastructure is having access to devices such as cellphones that do not need internet connectivity, which may therefore be more affordable and accessible to a wider user population. These phones can receive short messages containing patient health information or knowledge from healthcare organizations.

**Table 4:** Infrastructures and mechanisms for telehealth systems and services.

Infrastructure layer	Mechanisms	Service examples	Examples developing countries or LMICs and regions
Wearables, high-resolution cameras, cloud data storage, health information exchange, medical equipment, robots	Real-time and continuous health data syncing, remote control for real-time treatment.	• Remote monitoring systems for quarantined patients^49^. • Remote monitoring of blood pressure^92^. • Real-time teleretinal photocoagulation^27^.	• Urban China, Urban Brazil^97^
Devices with audio and cameras, and stable internet connection	Audio and video-based remote consultation, video conferencing	• Mobile application for remote dermatology diagnosis based on photos and videos sent by patients^51^.	• Saudi Arabia^98^, Pakistan^99^
Basic internet connection, computers/smartphones	Patient-initiated, text-based remote consultation; hotline	• Mental health hotline^52^.	• Rural China, Sri Lanka^100^
Cell phones with basic functions, Short Message Service (SMS)	Emergency notification and public education through short messages; making appointments	• Short messages for knowledge dissemination^48^. • Short messages for making and changing appointments^96^.	• Kenya^101^, Rwanda^102^

Moving beyond the basic level, the availability of internet connection, computers, and smartphones enables telehealth services like text-based remote consultations, through which patients can send symptoms and questions to providers and receive treatment recommendations.

Building upon this foundation, a more advanced infrastructure involves the integration of audios, cameras, and a stable internet connection. These technologies facilitate more versatile telehealth services with flexible modalities. For instance, specialties such as dermatology often necessitate photos and videos from patients to make accurate diagnoses, and text-based communication alone may not be sufficient. The inclusion of stable internet connections and video capabilities allows for remote, synchronous video conferencing, enabling interactive patient-provider communication and allowing clinicians to gather more comprehensive information.

The highest level of infrastructure encompasses wearables and cloud-based data storage, along with health information exchange capabilities. These components enable telehealth services that go beyond remote consultations. For instance, patients can use wearables to continuously collect health data such as vital signs. The patient-generated health data (PGHD) can be synchronized in real-time with providers, supporting clinical decision-making processes^62^. Furthermore, healthcare providers can also perform real-time remote treatments, such as surgeries and deep brain stimulation, through remote control^40^.

## Discussion

Telehealth played a vital role in supporting the continuity of care during the COVID-19 pandemic. It was especially crucial for developing countries or LIMICs where healthcare resources were limited and unequally distributed. While previous reports on experiences to implement and adopt telehealth during the pandemic offered valuable insights, they mostly focused on describing the systems that were adopted in their specific contexts rather than a holistic review of the process of how they were implemented and adopted^63,64^. To address this gap, our review, guided by the CFIR, identified barriers, successful experiences, and recommended practices related to the implementation and adoption of telehealth from multiple perspectives. These perspectives encompassed macro-level practices, such as leveraging social media to raise awareness about telehealth services at the societal level. We also examined technological features that supported flexible modalities of telehealth services. Additionally, we explored processes that facilitated individual uptake of telehealth, such as involving family caregivers to assist children’s or older adults’ use of telehealth services^65^. Further, we proposed a framework comprising layers of infrastructures to support various levels of telehealth services. This framework may serve as a useful tool for developing countries or LMICs with different infrastructural readiness to plan their telehealth implementation strategies.

This systematic review reported the lessons and evidence of telehealth implementation and adoption in China during the pandemic from different aspects as guided by the CFIR framework. The findings from different aspects of telehealth implementation provide crucial implications at various levels.

In China, telehealth systems have proven to be instrumental in enabling patients to consult specialists beyond their physical reach. By using these systems for screening and triaging patients, hospitals have successfully reduced in-person visits, curbed in-hospital infections, and eased the burden on healthcare workers—crucial achievements during the pandemic. Furthermore, China’s experiences highlight that telehealth systems can also contribute to equalizing medical resource distribution and preventing in-hospital infections^66^.

A key factor in the successful implementation of telehealth in China was the presence of a compatible technological infrastructure. While internet connectivity and smartphones supported common telehealth services like diagnosis, more advanced real-time treatments necessitated sophisticated infrastructure^66^. While such infrastructure may have been easily accessible and well-established in developed countries, they were not yet pervasive and affordable in most LMICs or resource-limited settings. Developing and maintaining such infrastructures may also be time-consuming and costly for LMICs. Therefore, policymakers and practitioners should make use of existing infrastructures to support immediate telehealth services and start strategic plans for long-term infrastructure development. In addition, developing countries have heterogeneous infrastructure readiness and need tailored solutions to successfully implement telehealth. In China, commercial apps and software, such as WeChat, played a significant role as telehealth platforms. However, other LMICs might find apps like WhatsApp more suitable due to their popularity and familiarity^67^. These social networking platforms, including WeChat, Zoom, WhatsApp, Facebook, and FaceTime in LMICs provided a low-barrier communication tool already widely used in healthcare settings for video calls, phone calls, voice messages, and the sharing of images and videos. The infrastructure model we recommended (Table 4) may serve as a pragmatic framework for practitioners in developing countries or LMICs to plan telehealth implementation based on their infrastructure readiness and telehealth service goals.

The multi-component and personalized nature of telehealth, coupled with the rapid pace of change in mobile technology and the context-specific application of these tools, presents various challenges. Another important factor in successfully implementing telehealth services in LMICs is having a prepared and well-trained team that includes not only healthcare professionals but also health IT personnel. During public health crises, the immediate responses to implement telehealth services are crucial. However, hospitals alone often are not adequate for such efforts that require intensive technical expertize and resources. Some studies highlighted the importance of collaborating with third-party technology companies or government-funded information centers to facilitate the implementation and maintenance of telehealth systems^43,55^. However, it is important to note that burdening healthcare providers with the responsibility of managing telehealth tasks, including assisting patients in using the system, may lead to burnout. Thus, engaging and training health IT professionals is necessary to provide adequate support to the telehealth system. China’s experience suggests the potential of the cooperation of healthcare organizations, government, non-government agencies, as well as technology companies and financial inputs from philanthropic agencies.

In developing countries or LMICs, where patients’ digital literacy tends to be lower, there is increased vulnerability to privacy issues when using telehealth services. A large portion of telehealth services reported in the included papers used available commercial technologies such as WeChat and Zoom, but few reported strategies to address potential privacy concerns. As telehealth systems collected, stored, and transmitted protected health information (PHI), any security breach could pose harm to patients. While telehealth services were available in many areas of China, there was no stringent regulatory authority to monitor the quality of these services and maintain ethical standards. To fully harness the benefits of telehealth while mitigating negative consequences, it is essential for China and other developing countries to develop policies that ensure the safety of patients’ privacy, especially those with compromised digital literacy. Establishing robust regulatory frameworks and promoting data protection measures will be crucial steps toward the responsible and secure expansion of telehealth services in these regions.

Adopting telehealth into clinical practice in developing countries or LMICs may be a means to lower costs and conserve resources in the long run, thereby alleviating the burden of out-of-pocket spending and boosting the population’s access to affordable health care. Particularly given that out-of-pocket payments for health care services make for a considerable share of overall health spending in many LMICs that have no general health insurance available. Several studies conducted in LMICs have demonstrated the cost-saving potential of telehealth services, as healthcare providers can offer online consultations at a lower cost compared to face-to-face visits, while also reducing transportation expenses when health institutions are distant^68,69^. Healthcare systems should establish incentive mechanisms, such as performance appraisal, social prestige, and financial bonuses to facilitate the adoption of telehealth.

In many developing countries, the digital divide is a constant concern that impacts the effectiveness and coverage of telehealth. For example, older populations, especially those with low income, need the most medical resources but are the least likely to have access to internet-based social networking services. In addition, they may find it difficult to use complicated apps on mobile phones^70,71^. To address this issue, researchers have recommended leveraging off-the-shelf technologies that are already widely accepted by the public, such as WeChat^37,72^. Additionally, family caregivers play a crucial role in assisting patients facing technological challenges^49^. To reach a diverse and wide population to disseminate knowledge and health information, it is preferable to choose technologies that presume low barriers and high accessibility such as SMS or phone calls. It is important to increase the awareness of telehealth services among the public, especially marginalized populations who may benefit more from telehealth services but are not aware of them. This awareness-building effort is especially critical for LMICs, where rural populations might not have sufficient exposure to telehealth services.

To achieve more relevant and responsive healthcare services, it is essential to expand access to these services and implement organizational reforms to attain “close-to-client” approaches^73^. Decentralization has been promoted as a means for improving the responsiveness and effectiveness of health systems in LMICs^74,75^. Telehealth enables the expansion of care away from a centralized location to primary care, communities, and into the home, with primary care provider groups integrating virtual care directly into their practice^76^. Telehealth makes it feasible to distribute medical resources to remote and rural areas by decentralizing resources and services to primary care clinics^77^. In LMICs, developing infrastructures and policies may be time-consuming and may not yield immediate benefits, especially during the pandemic. China’s experiences suggested that governments could support core hospitals (e.g., tertiary hospitals) in developing telehealth systems and providing services to lower-level hospitals (e.g., primary hospitals or community health care centers). Patients in rural and remote areas with limited medical resources can consult medical experts from core hospitals through telehealth systems and receive high-quality care without traveling. In the future, China and other developing countries should further explore how to best allocate the limited healthcare resources through telehealth systems.

A developing country or LMIC can make strategic choices to guide the implementation and adoption of telehealth systems. These strategies are directly linked to or limited by the country’s governance and technology readiness. Countries that have government-led healthcare systems and robust technological foundations, such as China and Rwanda^78^, could prioritize strengthening the national telehealth strategy by prioritizing telehealth implementation at the national level. Building a national telehealth ecosystem requires multiple enablers, including the healthcare-governance model (centralized versus decentralized and predominantly public versus private) and digital readiness as a key element in defining a country’s implementation pathway for telehealth. In the near term, governments can intervene directly by creating dedicated teams responsible and accountable for the efforts around telehealth implementation strategy. The long-term strategy for these countries is to develop mechanisms for sustainable financing of the telehealth system.

Developing countries or LMICs that were with a private sector-led health technology effort and strong digital foundations could prioritize telehealth implementation at a national level through strategic partnerships with the private sectors. To attract private-sector investment and ensure the viability of telehealth initiatives, these countries could also consider enacting telehealth legislation and policies. For instance, India benefits from a flourishing digital- healthcare private sector and strong country-level action to develop a national telehealth ecosystem^79^. In the long term, LMICs’ governments should work with solution providers to evaluate how to integrate with national or regional telehealth systems and may depend on strong, top-level government leaders to champion system change and drive user adoption. Partnering with established organizations, service providers, or government agencies is often vital to reaching scale.

Developing countries or LMICs with nascent technology readiness may need to prioritize the development and improvement of digital technology readiness by investing in infrastructure and capability building^80^. In the short term, the solution providers and governments should build interlocking relationships when deploying telehealth platforms. With government incentives, solution providers may start developing critical use cases such as health care payments to bypass the challenging financing mechanisms and remote care, to prove the concept and lay the foundation for a telehealth platform. In the long term, these LIMCs can also build a forward-looking, national strategy that demonstrates the government’s commitment to promoting telehealth.

This systematic review has some limitations. First, because many studies were published during the pandemic, which aimed to rapidly share evidence and experiences, the quality of the publications was relatively low; the study design for these publications was primarily observational in nature with modest methodological rigor. Prior research has indicated that COVID-19 research ranked low in the hierarchy of scientific evidence, with numerous articles published in lower impact factor journals and within a shorter timeframe^81^. The present review did not aim to assess the effect of the reviewed systems or the quality of the target studies. We sought to identify the properties of telehealth systems and recognize the state of telehealth systems or services in China during the COVID-19 pandemic. Second, the majority of studies were conducted in urban areas and major modern cities in China, where the population has high digital literacy and overall economic development that may not be fully representative of the nation as well as developing countries. Although we included articles that were published in Chinese, we acknowledge that it can be challenging for projects in less-developed regions in China to publish research articles, which could explain why many study sites were from major modern cities. However, the resources and infrastructures from the selected studies varied and could offer insights into different resource-limited settings. Third, the included studies may not be fully representative since most of them reported on telehealth services in the early stages of the pandemic. This might be because China altered its prevention and control policy after April 2020, and quarantine restrictions were relaxed after the first wave. As a result, the momentum for building new telehealth systems might have gradually decreased thereafter. Nevertheless, we compared the results with other studies^82,83^ and found that our findings were consistent with those from other LMICs, indicating that our results were representative. Fourth, the implications from China may be applicable to countries that are similar to China in terms of their economies, rural-urban divisions, and healthcare systems. What worked effectively in China may not be directly applicable or feasible in other countries due to variations in resources, technology level, and healthcare delivery models. Socio-cultural factors also influence healthcare practices and patient behavior. Approaches that were successful in China might not align with the cultural norms, beliefs, and preferences in other countries. Adapting telehealth practices to account for these factors is crucial to ensure acceptance and uptake. Future work should explore telehealth service delivery in rural areas in developing countries or LMICs to find best practices and practical solutions.

Through a systematic review of telehealth systems used in China during the COVID-19 pandemic, we summarized the characteristics of the infrastructures and identified the barriers and successful experiences in implementing the systems. Successful implementation strategies of telehealth systems necessitate collaborative efforts from various stakeholders, strong governmental support, tailored designs for marginalized populations, and strategic technological infrastructure development. The experiences and practices observed in China hold valuable insights and can serve as robust evidence for broader scaling in other developing countries and resource-limited settings.

### Reporting summary

Further information on research design is available in the Nature Research Reporting Summary linked to this article.

### Supplementary information

Supplementary Material

Reporting Summary

## Data Availability

All data are incorporated into the article and its online supplementary material.

## References

[1] Moynihan R (2021). Impact of COVID-19 pandemic on utilisation of healthcare services: a systematic review. BMJ open.

[2] World Health Organization, *Implementing telemedicine services during COVID-19: guiding principles and considerations for a stepwise approach*. (2020).

[3] Caffery LJ, Farjian M, Smith AC (2016). Telehealth interventions for reducing waiting lists and waiting times for specialist outpatient services: A scoping review. J. Telemed. telecare.

[4] Imlach F (2020). Telehealth consultations in general practice during a pandemic lockdown: survey and interviews on patient experiences and preferences. BMC Fam. Pract..

[5] Jnr BA (2020). Use of telemedicine and virtual care for remote treatment in response to COVID-19 pandemic. J. Med. Syst..

[6] Snoswell, C. L., et al. The clinical effectiveness of telehealth: a systematic review of meta-analyses from 2010 to 2019. *J. Telemed. Telecare*. 10.1177/1357633X211022907 (2021). 10.1177/1357633X211022907 34184580

[7] Wijesooriya NR (2020). COVID-19 and telehealth, education, and research adaptations. Paediatr. Respiratory Rev..

[8] Gajbhiye RK (2021). Differential impact of COVID‐19 in pregnant women from high‐income countries and low‐to middle‐income countries: A systematic review and meta‐analysis. Int. J. Gynecol. Obstet..

[9] Kitano T (2021). The differential impact of pediatric COVID-19 between high-income countries and low-and middle-income countries: A systematic review of fatality and ICU admission in children worldwide. PloS one.

[10] World Bank Group, *World Bank Country and Lending Groups 2020*. (2020).

[11] Zhang QF, Hu Z (2021). Rural China under the COVID‐19 pandemic: Differentiated impacts, rural–urban inequality and agro‐industrialization. J. Agrarian Change.

[12] population, W.B.U., *United Nations Population Division. World Urbanization Prospects: 2018 Revision*.

[13] Ling RE (2011). Emerging issues in public health: a perspective on China’s healthcare system. Public Health.

[14] Xiong Y, Huang J (2016). Inequality in health service between urban and rural areas in China: evidence from CHARLS 2013 data. Population Journal.

[15] Song S (2019). Increased inequalities in health resource and access to health care in rural China. Int. J. Environ. Res. public health.

[16] Ye, J. & Ren, Z. Examining the impact of sex differences and the COVID-19 pandemic on health and health care: findings from a national cross-sectional study. *JAMIA Open*, (2022). 10.1093/jamiaopen/ooac076 PMC9494404 36177395

[17] Koonin LM (2020). Trends in the use of telehealth during the emergence of the COVID-19 pandemic—United States, January–March 2020. Morbidity Mortal. Wkly. Rep..

[18] Ye J, Wang Z, Hai J (2022). Social Networking Service, Patient-Generated Health Data, and Population Health Informatics: National Cross-sectional Study of Patterns and Implications of Leveraging Digital Technologies to Support Mental Health and Well-being. J. Med. Internet Res..

[19] Page MJ (2021). The PRISMA 2020 statement: an updated guideline for reporting systematic reviews. Syst. Rev..

[20] Ye, J., He L., and Beestrum M., *Implications for implementation and adoption of telehealth in low- and middle-income countries during the COVID-19 pandemic: a systematic review of China’s practices and experiences. Available at*: https://www.crd.york.ac.uk/prospero/display_record.php?ID=CRD42023402844. 10.1038/s41746-023-00908-6 PMC10507083 37723237

[21] Khandkar SH (2009). Open coding. Univ. Calg..

[22] Damschroder LJ (2009). Fostering implementation of health services research findings into practice: a consolidated framework for advancing implementation science. Implement. Sci..

[23] Yearbook, C. S., *China Statistical Yearbook: the national bureau of statistics of the People’s Republic of China*. (2021).

[24] Xue, H., Shi, Y. & Medina, A. Who are rural China’s village clinicians? *China Agricultural Economic Review*, (2016).

[25] Cai H (2016). Application of telemedicine in Gansu Province of China. PLoS One.

[26] Han Y (2021). The use of remote programming for spinal cord stimulation for patients with chronic pain during the COVID-19 outbreak in China. Neuromodulation: Technol. Neural Interface.

[27] Chen H (2021). Application of 5G Technology to Conduct Real-Time Teleretinal Laser Photocoagulation for the Treatment of Diabetic Retinopathy. JAMA Ophthalmol..

[28] Li P (2020). How telemedicine integrated into China’s anti-COVID-19 strategies: case from a National Referral Center. BMJ Health Care Inform.

[29] Wang Y (2021). Application of telemedicine in the COVID-19 epidemic: An analysis of Gansu Province in China. Plos one.

[30] Zhai Y (2021). An internet-based multidisciplinary online medical consultation system to help cope with pediatric medical needs during the COVID-19 outbreak: a cross-sectional study. Transl. Pediatrics.

[31] Fu Z (2021). Minimizing the Impact of the COVID-19 Epidemic on Oncology Clinical Trials: Retrospective Study of Beijing Cancer Hospital. J. Med. Internet Res..

[32] Dorsey ER, Topol EJ (2016). State of telehealth. N. Engl. J. Med..

[33] Liu L (2020). Application and preliminary outcomes of remote diagnosis and treatment during the COVID-19 outbreak: retrospective cohort study. JMIR mHealth uHealth.

[34] Li H-L (2020). Pilot study using telemedicine video consultation for vascular patients’ care during the COVID-19 period. Ann. Vasc. Surg..

[35] Zhang J (2020). Application value of vital signs telemetry system for 2019 novel coronavirus disease suspected cases in isolation wards. Infect. Drug Resistance.

[36] Lu Y (2020). Management of intractable pain in patients with implanted spinal cord stimulation devices during the COVID-19 pandemic using a remote and wireless programming system. Front. Neurosci..

[37] Ku BPS (2021). Tele-Rehabilitation to Combat Rehabilitation Service Disruption During COVID-19 in Hong Kong: Observational Study. JMIR Rehabilitation Assistive Technol..

[38] Zhou F (2020). Online clinical consultation as a utility tool for managing medical crisis during a pandemic: retrospective analysis on the characteristics of online clinical consultations during the COVID-19 pandemic. J. Prim. Care Community Health.

[39] Lee AK (2020). Mitigation of head and neck cancer service disruption during COVID‐19 in Hong Kong through telehealth and multi‐institutional collaboration. Head. Neck.

[40] Lin Z (2020). Deep brain stimulation telemedicine programming during the COVID-19 pandemic: treatment of patients with psychiatric disorders. Neurosurgical focus.

[41] Ye, J., Hai, J., Song, J., Wang, Z. Multimodal Data Hybrid Fusion and Natural Language Processing for Clinical Prediction Models. medRxiv. 2023–08. 10.1101/2023.08.24.23294597 (2023). PMC11141806 38827058

[42] Ye, J. and L. N. Sanchez-Pinto. Three data-driven phenotypes of multiple organ dysfunction syndrome preserved from early childhood to middle adulthood. in *AMIA Annual Symposium Proceedings*. American Medical Informatics Association (2020). PMC8075454 33936511

[43] He Q (2021). Practice in Information Technology Support for Fangcang Shelter Hospital during COVID-19 Epidemic in Wuhan, China. J. Med. Syst..

[44] Ye, J. et al. & Hypertension Treatment in Nigeria Program investigators. Optimizing Longitudinal Retention in Care Among Patients With Hypertension in Primary Healthcare Settings: Findings From the Hypertension Treatment in Nigeria Program. *Circulation* **146**, A13217 (2022).

[45] Guo Z (2021). The safety and feasibility of the screening for retinopathy of prematurity assisted by telemedicine network during COVID-19 pandemic in Wuhan, China. BMC Ophthalmol..

[46] Ye J (2023). Patient Safety of Perioperative Medication Through the Lens of Digital Health and Artificial Intelligence. JMIR Perioperative Med..

[47] Ye J (2022). Characteristics and Patterns of Retention in Hypertension Care in Primary Care Settings From the Hypertension Treatment in Nigeria Program. JAMA Netw. Open.

[48] Wei, X., et al., Evaluation on application of intelligent voice call system in popularizing knowledge of prevention and control of COVID-19 for chronic disease patients in the community. *Chinese Journal of General Practitioners*, p. 388-393, (2020).

[49] Lee, S. H., et al. Establish a Real-time Responsible Home Quarantine and Monitoring Management mHealth Platform. in *AMIA Annual Symposium Proceedings*. American Medical Informatics Association (2020). PMC8075449 33936444

[50] Ye J (2017). A portable urine analyzer based on colorimetric detection. Anal. Methods.

[51] Mu Z (2021). Teledermatology Service During the COVID-19 Pandemic in China: A Mobile Application-Based Retrospective Study. Clin., Cosmet. investigational Dermatol..

[52] Ma R, Nguyen R, Oakman JM (2020). Dissemination strategies and usage of psychological assistance hotlines during the COVID-19 outbreak in China. Frontiers in Communication.

[53] Ye J (2020). Pediatric Mental and Behavioral Health in the Period of Quarantine and Social Distancing With COVID-19. JMIR pediatrics Parent..

[54] Ye J (2021). Advancing mental health and psychological support for health care workers using digital technologies and platforms. JMIR Formative Res..

[55] Lian W (2020). Digital health technologies respond to the COVID-19 pandemic in a tertiary hospital in China: development and usability study. J. Med. Internet Res..

[56] Zhang W (2022). Analyzing National Telemedicine Policies in China from the perspective of Policy Instrument (1997-2020). Int. J. Med. Inform..

[57] Wong, D., China’s Healthcare Industry: Opportunities in Telemedicine and Digital Healthcare. *China Briefing*, (2020).

[58] BAARK E (2022). China’s New Digital Infrastructure: Expanding 5G Mobile Communications. East Asian Policy.

[59] Helou S (2020). The effect of the COVID-19 pandemic on physicians’ use and perception of telehealth: The case of Lebanon. Int. J. Environ. Res. public health.

[60] Ye J (2020). The role of health technology and informatics in a global public health emergency: practices and implications from the COVID-19 pandemic. JMIR Med. Inform..

[61] Ye J (2020). Identifying Practice Facilitation Delays and Barriers in Primary Care Quality Improvement. J. Am. Board Fam. Med.: JABFM.

[62] Ye, J., The impact of electronic health record–integrated patient-generated health data on clinician burnout. *Journal of the American Medical Informatics Association*, (2021). 10.1093/jamia/ocab017 PMC8068436 33822095

[63] Doraiswamy S (2020). Use of telehealth during the COVID-19 pandemic: scoping review. J. Med. Internet Res..

[64] Monaghesh E, Hajizadeh A (2020). The role of telehealth during COVID-19 outbreak: a systematic review based on current evidence. BMC public health.

[65] Ye J (2023). Leveraging natural language processing and geospatial time series model to analyze COVID-19 vaccination sentiment dynamics on Tweets. JAMIA Open.

[66] Ye J (2021). Health Information System’s Responses to COVID-19 Pandemic in China: A National Cross-sectional Study. Appl. Clin. Inform..

[67] Barayev E (2021). WhatsApp Tele-Medicine–usage patterns and physicians views on the platform. Isr. J. health policy Res..

[68] Elhadi M (2021). Utilization of Telehealth Services in Libya in Response to the COVID-19 Pandemic: Cross-sectional Analysis. JMIR. Med. Inform..

[69] Shalash A (2020). Adopting virtual visits for Parkinson’s disease patients during the COVID-19 pandemic in a developing country. Front. Neurol.

[70] Ginsburg OM (2014). An mHealth model to increase clinic attendance for breast symptoms in rural Bangladesh: can bridging the digital divide help close the cancer divide?. oncologist.

[71] Ye, J. and Q. Ma. The effects and patterns among mobile health, social determinants, and physical activity: a nationally representative cross-sectional study. in *AMIA Annual Symposium Proceedings*. American Medical Informatics Association (2021). PMC8378627 34457181

[72] Liu G (2021). The efficacy of WeChat-Based parenting training on the psychological well-being of mothers with children with autism during the COVID-19 Pandemic: Quasi-experimental study. JMIR Ment. Health.

[73] Bank, W., *World development report 2004: making services work for poor people*.: The World Bank (2003).

[74] Guanais FC, Macinko J (2009). The health effects of decentralizing primary care in Brazil. Health Aff..

[75] Reddy TSK (2021). Decentralization of India Hypertension Control Initiative services to maintain continuum of care for hypertensive patients during COVID-19 pandemic in Telangana. WHO South-East Asia J. Public Health.

[76] Ye J (2022). Identifying Contextual Factors and Strategies for Practice Facilitation in Primary Care Quality Improvement Using an Informatics-Driven Model: Framework Development and Mixed Methods Case Study. JMIR Hum. Factors.

[77] Ye, J. Design and development of an informatics-driven implementation research framework for primary care studies. in *AMIA Annual Symposium Proceedings*. American Medical Informatics Association (2021). PMC8861697 35308925

[78] Dzinamarira T (2021). COVID-19: Comparison of the Response in Rwanda, South Africa and Zimbabwe. MEDICC Rev..

[79] Agarwal N (2020). Telemedicine in India: A tool for transforming health care in the era of COVID-19 pandemic. J. Educ. Health Promotion.

[80] Ye J (2020). Predicting mortality in critically ill patients with diabetes using machine learning and clinical notes. BMC Med. Inform. Decis. Mak..

[81] Jung RG (2021). Methodological quality of COVID-19 clinical research. Nat. Commun..

[82] Eslami Jahromi M, Ayatollahi H (2023). Utilization of telehealth to manage the Covid-19 pandemic in low-and middle-income countries: a scoping review. J. Am. Med. Inform. Assoc.

[83] Mahmoud, K., C. Jaramillo, and S. Barteit, Telemedicine in low-and middle-income countries during the COVID-19 pandemic: a scoping review. *Frontiers in Public Health*, **10**, 914423 (2022). 10.3389/fpubh.2022.914423 PMC9257012 35812479

[84] Xu H (2020). Monitoring and management of home-quarantined patients with COVID-19 using a WeChat-based telemedicine system: retrospective cohort study. J. Med. Internet Res..

[85] Shao C (2020). Taizhou’s COVID-19 prevention and control experience with telemedicine features. Front. Med..

[86] Chen M (2020). Characteristics of online medical care consultation for pregnant women during the COVID-19 outbreak: cross-sectional study. BMJ open.

[87] Zhang Q-L (2021). Telemedicine usage via WeChat for children with congenital heart disease preoperatively during COVID-19 pandemic: a retrospective analysis. Int. J. Qual. Health Care.

[88] Nan J (2020). Comparison of clinical outcomes in patients with ST elevation myocardial infarction with percutaneous coronary intervention and the use of a telemedicine app before and after the COVID-19 pandemic at a center in Beijing, China, from August 2019 to March 2020. Med. Sci. Monit.: Int. Med. J. Exp. Clin. Res..

[89] Li H (2021). The Establishment and Practice of Pharmacy Care Service Based on Internet Social Media: Telemedicine in Response to the COVID-19 Pandemic. Front. Pharmacol..

[90] Li Z (2021). Innovative Strategies and Efforts of Clinical Pharmacy Services During and After COVID-19 Epidemic: Experience from Shanghai Children’s Hospital. Risk Manag. Healthc. Policy.

[91] Ding L (2020). The internet hospital plus drug delivery platform for health management during the COVID-19 pandemic: observational study. J. Med. Internet Res..

[92] Zhang S (2021). Changes in home blood pressure monitored among elderly patients with hypertension during the covid-19 outbreak: a longitudinal study in China leveraging a smartphone-based application. Circulation: Cardiovascular Qual. Outcomes.

[93] Xia, W., et al., A telerehabilitation programme in post-discharge COVID-19 patients (TERECO): a randomised controlled trial. *Thorax*, (2021). 10.1136/thoraxjnl-2021-217382 PMC8318721 34312316

[94] Zhang C (2021). Utility of deep brain stimulation telemedicine for patients with movement disorders during the COVID‐19 outbreak in China. Neuromodulation: Technol. Neural Interface.

[95] Li L (2021). Management of follow-up with preterm infants during the outbreak in China. Front. Pediatrics.

[96] Lai TH (2021). The use of short message service (SMS) to reduce outpatient attendance in ophthalmic clinics during the coronavirus pandemic. Int. Ophthalmol..

[97] Caetano R (2020). Challenges and opportunities for telehealth during the COVID-19 pandemic: ideas on spaces and initiatives in the Brazilian context. Cad. de. Saúde. Pública.

[98] Alghamdi SM, Alqahtani JS, Aldhahir AM (2020). Current status of telehealth in Saudi Arabia during COVID-19. J. Fam. community Med..

[99] Nadeem T, Siddiqui S, Asad N (2020). Initiating psychiatry teleclinics during the COVID-19 pandemic in a tertiary care hospital in Karachi, Pakistan. Psychological Trauma.: Theory, Res., Pract., Policy.

[100] Kulatunga G (2020). A review of Telehealth practices in Sri Lanka in the context of the COVID-19 pandemic. Sri Lanka J. Bio-Med. Inform..

[101] Lester RT (2010). Effects of a mobile phone short message service on antiretroviral treatment adherence in Kenya (WelTel Kenya1): a randomised trial. Lancet.

[102] El Joueidi S (2021). Evaluation of the implementation process of the mobile health platform ‘WelTel’in six sites in East Africa and Canada using the modified consolidated framework for implementation research (mCFIR). BMC Med. Inform. Decis. Mak..

