# Supplementary material for: Implications for implementation and adoption of telehealth in developing countries: a systematic review of China’s practices and experiences
Source: NPJ Digit Med. 2023 Sep 18;6:174. doi: 10.1038/s41746-023-00908-6 (PMC10507083; doi:10.1038/s41746-023-00908-6)
Supplement: Supplementary file 1 — Supplementary Material [file 41746_2023_908_MOESM1_ESM.pdf]

Supplementary Table 1. Search strategies for the study

Database	Search strategy
PubMed/ MEDLINE	Wireless technology[MeSH] OR "Smartphone"[MeSH] OR "Telephone"[MeSH] OR "Telemedicine"[MeSH] OR "Text Messaging"[Mesh] OR "Cell Phone"[Mesh] OR "Cell Phone Use"[Mesh] OR Telemedicine*[tiab] OR Tele-medicine*[tiab] OR Tele-health[tiab] OR Telehealth[tiab] OR Smart-phone*[tiab] OR Smartphone*[tiab] OR Remote-consult*[tiab] OR Video-call*[tiab] OR Video-consult*[tiab] OR Wireless-technolog*[tiab] OR Video-communication*[tiab] OR Wireless-system*[tiab] OR Videoconferenc*[tiab] OR Video-conferenc*[tiab] OR E-health[tiab] OR M-health[tiab] OR Ehealth[tiab] OR Mhealth[tiab] OR Cellular-phone*[tiab] OR Cell-phone*[tiab] OR Cellular-Telephone*[tiab] OR Mobile-Phone*[tiab] OR Mobile-Telephone*[tiab] OR Tele-conferenc*[tiab] OR Digital-health[tiab] OR Video-visit*[tiab] OR Video-call*[tiab] OR Tele-consult*[tiab] OR Telehealthcare*[tiab] OR Tele-monitor*[tiab] OR Remote-monitor*[tiab] OR Remote-diagnos*[tiab] OR Tele-diagnos*[tiab] OR telecommunication*[tiab] OR Texting*[tiab] OR Short-Message*[tiab] OR Text-messag*[tiab] OR SMS-messag*[tiab] OR Multimedia-messag*[tiab] OR MMS-messag*[tiab] OR Mobile-reminder*[tiab] OR app[tiab] OR apps[tiab] OR Mobile-application*[tiab] OR Mobile-based[tiab] OR phone[tiab] OR telephone[tiab] OR bluetooth[tiab] OR wireless[tiab] OR mobile-device*[tiab] OR personal-digital-assistant*[tiab] OR PDA[tiab] OR PDAs[tiab] OR patient-monitoring-device*[tiab] OR HealthCall[tiab] OR GPRS[tiab] OR 3G[tiab] OR 4G[tiab] OR 5G[tiab] OR global-positioning-system*[tiab] OR GPS[tiab] OR "interactive voice response"[tiab]<br>AND<br>China[Mesh] OR china*[tiab] OR chinese*[tiab] OR hong-kong*[tiab] OR macau*[tiab]<br>AND<br>COVID-19[Mesh] OR "COVID-19 Testing"[Mesh] OR "SARS-CoV-2"[Mesh] OR "COVID-19 Vaccines"[Mesh] OR Coronavirus[mesh:noexp] OR coronavirus infections[mesh:noexp] OR COVID[tiab] OR COVID19[tiab] OR coronavirus*[tiab] OR "corona virus*"[tiab] OR nCOV[tiab] OR 2019nCOV[tiab] OR COV[tiab] OR COV2[tiab] OR SARSCOV*[tiab] OR SARS2[tiab]
Embase	'wireless technology'/exp OR 'wireless technology':ti,ab OR 'smartphone'/exp OR smartphone:ti,ab OR 'telephone'/exp OR telephone:ti,ab OR 'telemedicine'/exp OR telemedicine:ti,ab OR 'text messaging'/exp OR 'text messaging':ti,ab OR 'cell phone'/exp OR 'cell phone':ti,ab OR 'cell phone use'/exp OR 'cell phone use':ti,ab OR telemedicine*:ti,ab OR 'tele medicine*':ti,ab OR 'tele health':ti,ab OR telehealth:ti,ab OR 'smart phone*':ti,ab OR smartphone*:ti,ab OR 'remote consult*':ti,ab OR 'video consult*':ti,ab OR 'wireless technolog*':ti,ab OR 'video communication*':ti,ab OR 'wireless system*':ti,ab OR videoconferenc*:ti,ab OR 'video conferenc*':ti,ab OR 'e health':ti,ab OR 'm health':ti,ab OR ehealth:ti,ab OR mhealth:ti,ab OR 'cellular phone*':ti,ab OR 'cell phone*':ti,ab OR 'cellular telephone*':ti,ab OR 'mobile phone*':ti,ab OR 'mobile telephone*':ti,ab OR 'tele conferenc*':ti,ab OR 'digital health':ti,ab OR 'video visit*':ti,ab OR 'video call*':ti,ab OR 'tele consult*':ti,ab OR telehealthcare*:ti,ab OR 'tele monitor*':ti,ab OR 'remote monitor*':ti,ab OR 'remote diagnos*':ti,ab OR 'tele diagnos*':ti,ab OR telecommunication*:ti,ab OR texting*:ti,ab OR 'short message*':ti,ab OR 'text messag*':ti,ab OR 'sms messag*':ti,ab OR 'multimedia messag*':ti,ab OR 'mms messag*':ti,ab OR 'mobile reminder*':ti,ab OR app:ti,ab OR apps:ti,ab OR 'mobile application*':ti,ab OR 'mobile based':ti,ab OR bluetooth:ti,ab OR wireless:ti,ab OR 'mobile device*':ti,ab OR 'personal digital assistant*':ti,ab OR pda:ti,ab OR pdas:ti,ab OR 'patient monitoring device*':ti,ab OR healthcall:ti,ab OR gprs:ti,ab OR 3g:ti,ab OR 4g:ti,ab OR 5g:ti,ab OR 'global positioning system*':ti,ab OR gps:ti,ab OR 'interactive voice response':ti,ab<br>AND<br>'china'/exp OR china*:ti,ab OR chinese*:ti,ab OR 'hong kong*':ti,ab OR macau*:ti,ab<br>AND<br>'covid 19'/exp OR 'covid 19':ti,ab OR 'covid-19 testing'/exp OR 'covid-19 testing':ti,ab OR 'sars cov 2'/exp OR 'sars cov 2':ti,ab OR 'covid-19 vaccines'/exp OR 'covid-19 vaccines':ti,ab OR 'coronavirus'/exp OR coronavirus:ti,ab OR 'coronavirus infections'/exp OR 'coronavirus infections':ti,ab OR covid:ti,ab OR covid19:ti,ab OR coronavirus*:ti,ab OR 'corona virus*':ti,ab OR ncov:ti,ab OR 2019ncov:ti,ab OR cov:ti,ab OR cov2:ti,ab OR sarscov*:ti,ab OR sars2:ti,ab
Scopus	TITLE-ABS (telemedicine* OR tele-medicine* OR tele-health OR telehealth OR smart-phone* OR smartphone* OR remote-consult* OR video-call* OR video-consult* OR wireless-technolog* OR video-communication* OR wireless-system* OR videoconferenc* OR video-conferenc* OR e-health OR m-health OR ehealth OR mhealth OR cellular-phone* OR cell-phone* OR cellular-telephone* OR mobile-phone* OR mobile-telephone* OR tele-conferenc* OR digital-health OR video-visit* OR video-call* OR tele-consult* OR telehealthcare* OR tele-monitor* OR remote-monitor* OR remote-diagnos* OR tele-diagnos* OR telecommunication* OR texting* OR short-message* OR text-messag* OR sms-messag* OR multimedia-messag* OR mms-messag* OR mobile-reminder* OR app OR apps OR mobile-application* OR mobile-based OR phone OR telephone OR bluetooth OR wireless OR mobile-device* OR personal-digital-assistant* OR pda OR pdas OR patient-monitoring-device* OR healthcall OR gprs OR 3g OR 4g OR 5g OR global-positioning-system* OR gps OR "interactive voice response")<br>AND<br>TITLE-ABS (china* OR chinese OR "hong kong" OR macau*)<br>AND

	TITLE-ABS (covid OR covid-19 OR covid19 OR coronavirus* OR "corona virus*" OR ncov OR 2019ncov OR cov OR cov2 OR sarscov* OR sars2)
Cochrane Library	MeSH descriptor: [China] explode all trees OR china:ti,ab OR chinese:ti,ab OR "hong kong":ti,ab OR macau*:ti,ab<br>AND<br>MeSH descriptor: [COVID-19] explode all trees OR MeSH descriptor: [COVID-19 Testing] explode all trees OR MeSH descriptor: [SARS-CoV-2] explode all trees OR MeSH descriptor: [COVID-19 Vaccines] explode all trees OR MeSH descriptor: [Coronavirus] this term only OR MeSH descriptor: [Coronavirus Infections] this term only OR (COVID OR COVID19 OR coronavirus* OR "corona virus*" OR nCOV OR 2019nCOV OR COV OR COV2 OR SARSCOV* OR SARS2):ti,ab<br>AND<br>MeSH descriptor: [Wireless Technology] explode all trees OR MeSH descriptor: [Smartphone] explode all trees OR MeSH descriptor: [Telephone] explode all trees OR MeSH descriptor: [Telemedicine] explode all trees OR MeSH descriptor: [Text Messaging] explode all trees OR MeSH descriptor: [Cell Phone] explode all trees OR MeSH descriptor: [Cell Phone Use] explode all trees OR (Telemedicine*:ti,ab OR Tele-medicine*:ti,ab OR Tele-health:ti,ab OR Telehealth:ti,ab OR Smart-phone*:ti,ab OR Smartphone*:ti,ab OR Remote-consult*:ti,ab OR Video-call*:ti,ab OR Video-consult*:ti,ab OR Wireless-technolog*:ti,ab OR Video-communication*:ti,ab OR Wireless-system*:ti,ab OR Videoconferenc*:ti,ab OR Video-conferenc*:ti,ab OR E-health:ti,ab OR M-health:ti,ab OR Ehealth:ti,ab OR Mhealth:ti,ab OR Cellular-phone*:ti,ab OR Cell-phone*:ti,ab OR Cellular-Telephone*:ti,ab OR Mobile-Phone*:ti,ab OR Mobile-Telephone*:ti,ab OR Tele-conferenc*:ti,ab OR Digital-health:ti,ab OR Video-visit*:ti,ab OR Video-call*:ti,ab OR Tele-consult*:ti,ab OR Telehealthcare*:ti,ab OR Tele-monitor*:ti,ab OR Remote-monitor*:ti,ab OR Remote-diagnos*:ti,ab OR Tele-diagnos*:ti,ab OR telecommunication*:ti,ab OR Texting*:ti,ab OR Short-Message*:ti,ab OR Text-messag*:ti,ab OR SMS-messag*:ti,ab OR Multimedia-messag*:ti,ab OR MMS-messag*:ti,ab OR Mobile-reminder*:ti,ab OR app:ti,ab OR apps:ti,ab OR Mobile-application*:ti,ab OR Mobile-based:ti,ab OR phone:ti,ab OR telephone:ti,ab OR bluetooth:ti,ab OR wireless:ti,ab OR mobile-device*:ti,ab OR personal-digital-assistant*:ti,ab OR PDA:ti,ab OR PDAs:ti,ab OR patient-monitoring-device*:ti,ab OR HealthCall:ti,ab OR GPRS:ti,ab OR 3G:ti,ab OR 4G:ti,ab OR 5G:ti,ab OR global-positioning-system*:ti,ab OR GPS:ti,ab OR "interactive voice response":ti,ab)
Web of Science	"Wireless technology" OR Smartphone OR Telephone OR Telemedicine OR "Text Messaging" OR "Cell Phone" OR "Cell Phone Use" OR Telemedicine* OR Tele-medicine* OR Tele-health OR Telehealth OR Smart-phone* OR Smartphone* OR Remote-consult* OR Video-call* OR Video-consult* OR Wireless-technolog* OR Video-communication* OR Wireless-system* OR Videoconferenc* OR Video-conferenc* OR E-health OR M-health OR Ehealth OR Mhealth OR Cellular-phone* OR Cell-phone* OR Cellular-Telephone* OR Mobile-Phone* OR Mobile-Telephone* OR Tele-conferenc* OR Digital-health OR Video-visit* OR Video-call* OR Tele-consult* OR Telehealthcare* OR Tele-monitor* OR Remote-monitor* OR Remote-diagnos* OR Tele-diagnos* OR telecommunication* OR Texting* OR Short-Message* OR Text-messag* OR SMS-messag* OR Multimedia-messag* OR MMS-messag* OR Mobile-reminder* OR app OR apps OR Mobile-application* OR Mobile-based OR phone OR telephone OR bluetooth OR wireless OR mobile-device* OR personal-digital-assistant* OR PDA OR PDAs OR patient-monitoring-device* OR HealthCall OR GPRS OR 3G OR 4G OR 5G OR global-positioning-system* OR GPS OR "interactive voice response" (Topic)<br>AND<br>China OR china* OR chinese* OR hong-kong* OR macau* (Topic)<br>AND<br>COVID-19 OR "COVID-19 Testing" OR SARS-CoV-2 OR "COVID-19 Vaccines" OR Coronavirus OR "coronavirus infections" OR COVID OR COVID19 OR coronavirus* OR "corona virus*" OR nCOV OR 2019nCOV OR COV OR COV2 OR SARSCOV* OR SARS2 (Topic)

Supplementary Table 2. Summary of included telehealth articles with details (n = 36)

First author	Technology/platform	Target disease	Service	Infrastructure	Clinical outcome	Implementation outcome
Ku[1]	HIS, mobile application	General health	Rehabilitation via prescribe exercise videos and reminders to patients	Existing EHR; smartphone	Multiple	Utilization: Over 50% of the workforce prescribed telehealth; Adherence: 81.7%; Satisfaction: 3.7/5 for therapists and 4.2/5 for patients
He[2]	Cloud-based hospital information system	Non-critically ill patients with COVID19	Basic functions of isolation, triage, basic medical care, frequent monitoring and rapid referral, and essential living and social engagement; HIS provided two ways for patient registration after admittance—spreadsheet import and manual input.	-Internet connection	Total infection decreased 69.6%	Utilization: Almost 800 doctors, nurses, and lab staff have used
Guo[3]	WeChat	Retinopathy of prematurity	Screening	-Internet, smartphone	/	Utilization: 86 screening appointments were received, among which 67 (77.9%) were from telemedicine platform online.
Fu[4]	Data processing and application platform (DPAP)	Provide support to conduct anticancer drug trials	Supporting clinical trials	The hospital's clinical trial management system (CTMS); a remote monitoring system that integrates various business systems of the hospital under a virtual private network (VPN).	/	Effectiveness: 572 trials conducted with a protocol compliance rate of 85.24% for 3718 participants; 0 infection rate and error rate. Utilization: 176 clinical research associates from 76 sponsors or clinical research organizations had used the remote monitoring system to monitor 1318 participants in 228 trials conducted in 16 departments; approximately 40% of clinical trials were remotely monitored; the total number of log-ins was 10,470
Lian[5]	Wechat	/	Web-based consultation; psychological counseling; COVID-19 screening; COVID-19 related symptoms monitoring; COVID-19 knowledge dissemination	Internet, smartphone	/	Utilization: 96,642 people used the automated COVID-19 screening and symptom monitoring services 161,884 and 7,795,194 times, respectively
Wei[6]	An intelligent voice call system	Covid-19	Popularization of scientific knowledge of COVID-19 and protective measures.	-Call<br>-SMS<br>-Cellphone	/	Effectiveness: The patients' score to answer the popular science knowledge and preventive measures was statistically increased, 94.8% (235/248) of patients' knowledge of COVID-19 mainly came from voice call. Utilization: A total of 98,487 voice outbound calls were effectively connected, 141,201 messages were sent, with a read rate of 97.8% (141 201/144 405); Satisfaction:90.8%
Lu[7]	A remote programming system	Chronic intractable pain	Video-based real-time programming and palliative medicine for pain patients with a spinal cord stimulation implant.	Internet, smartphone	improvement was achieved with programming adjustment in 12 of 13 (92.3%) cases	Utilization:34 sessions of remote programming were conducted with 16 patients; satisfaction: 11 of the 16 (68.8%) patients reported that the system was user-friendly and met their needs
Lee[8]	Zoom, hospital app	Head and neck cancer	Consultations by doctors and nurses, outpatient wound care, palliative care at home, and academic meetings or multidisciplinary tumor board meetings.	Internet, computer, mobile phone	/	Effectiveness: Alleviating the number of pending head and neck cancer operations by 20%; head and neck elective operation sessions have been decreased to a third of its original number, and with an ongoing influx of new cases, the waiting time has been lengthened to as long as 6 months.
Chen[9]	Yue Yi Tong (a free online communication platform)	Obstetric care	Online medical care consultation for pregnant women: (1) routine antenatal check-up (reports of examination, appointments for antenatal check-up, method and time of delivery, and hospitalization process; (2) obstetric care-seeking behaviors (cancel or postpone scheduled ANC visits; change method or time of delivery); (3) abnormal symptoms (vaginal bleeding, abnormal fetal movement and abdominal pain); (4) maternal	Internet, smartphone	/	Utilization: 957 participants completed the satisfaction survey and 77.95% of the participants used the service for the first time; Satisfaction: 94.63% of the respondents

			comorbidities and pregnancy complications (gestational diabetes mellitus, hypertension disorders and hypothyroidism); and (5) other needs of e-health (remote fetal heart monitoring, electronic prescription and online pharmacies).			
Zhai[10]	WeChat	Pediatric disease	Pediatric medical services	Internet, mobile phone	Multiple	The response rate of experts to requests for online consultations was 100%. Utilization: The online visits accounted for 14.7% of all visits. Online patients came from all over the country, covering 91% of the administrative regions of China, and the largest proportion of inquiries were for infants (33.7%). 266 experts from 25 pediatric specialties completed 12,318 effective consultations
Liu[11]	WeChat	Autism spectrum disorder	Parenting training	Internet, mobile phone	Anxiety, Depression, Parenting Stress, Hope	Utilization: 40.0% logged their progress in home training each week and 61.5% logged their progress more than 80% of the time for all 20 weeks.; satisfaction: 90.4%
Zhang[12]	WeChat	Congenital heart disease	Education, and the question-and-answer service	Internet, mobile phone	Infants' physical condition; parents' depression and anxiety	Utilization: all participants
Zhang[13]	A smartphone-based app, WeChat	Hypertension	Home blood pressure measurement	Internet, smartphone, home blood pressure monitoring device	Systolic blood pressure	Utilization: Wuhan patients were more likely to check their BP via the app, while doctors were less likely to monitor the app for BP control during the pandemic.
Wong[14]	Omaha system (a comprehensive assessment tool used widely to help nurses to identify client needs and problems in environmental, psychosocial, physiological, and health-related behavior); WhatsApp	/	Case management from a nurse supported by a social service team via telephone call and weekly video messages covering self-care topics delivered via smartphone	Telephone	Activities of daily living, Medication adherence, Quality of life, Depression	Utilization: all participants
Xu[15]	WeChat	COVID-19	Monitoring the progression of home-quarantined patients with COVID-19	Internet, smartphone	Initial symptoms, White blood cell count, Lymphocyte count	Utilization: all participants
Li[16]	Telephone and WeChat	Multiple	Follow-up service after discharge	Internet, Smartphone	Prognosis of preterm infants; anxiety level of families	Satisfaction: 96.8% of parents were satisfied with online follow-up and 95.2% of parents thought that online follow-up had answered all their questions.
Nan[17]	Tiantanzhixin app	ST segment elevation myocardial infarction (STEMI)	All the patients can communicate with doctors via the app anytime using voice messages, text messages, or pictures. Trained professionals will answer the patients' questions.	Internet, smartphone	Major adverse cardiac event (MACE), all-causes of death, cardiac death, non-fatal myocardial infarction, stroke, any revascularization, definite or probable stent thrombosis, and new renal replacement therapy	Effectiveness: reduced pre- and post-hospital delay times in patients with STEMI, which resulted in the diminished fear of cross infection among the STEMI patients and better preparation after their arrival to reduce waiting and screening protocol times.
Liu[18]	WeChat		Online inquiry, consultation, and suggestions were provided to patients for concerns related to COVID-19. Offline noncontact drug delivery services following online ordering and payment.	Internet, smartphone	/	Utilization: all participants; Satisfaction: 98.1% of survey respondents

Li[19]	WeChat	Vascular disease	The vascular surgeons reviewed each patient's electronic medical record, telephoned the patients using a voice telephone call, explaining to the patients that a video call for follow-up of their vascular conditions in a virtual telemedicine clinic setting; telemedicine Video Call.	Internet, Smartphone	/	Effectiveness: Vascular doctors could offer advice better than a primary health care physician based on the reported symptoms and images seen over the video conference; an average time of 11 minutes per visit seems quite low for patients with complex vascular issues; Utilization: all participants; Satisfaction: all participants
Li[20]	WeChat	Multiple	Online free pharmaceutical consultations	Internet, Smartphone	/	Utilization: 1,432 views and 66 followers and completed 39 counseling cases in 2 months; Effectiveness: All consultations were completed within 4 h, and the completion rate was 100%; Satisfaction: 97.4%
Li[21]	TERECO platform	COVID-19	Telerehabilitation for COVID-19 survivors	Internet, smartphone	Exercise capacity, lower limb muscle strength (LMS), pulmonary function, health-related quality of life (HRQOL) and dyspnea.	Effectiveness: The TERECO programme was effective in functional exercise capacity, lower limb muscle strength, and health-related quality of life. Utilization: all participants; Satisfaction: all participants
Li[22]	Huayitong platform	General health	Triage, consultations, education, rounds, CT scanning, treatment and follow-ups.	Internet, smartphone	/	Utilization: 10557 online consultations; only 8.1% (447 of 5517) of physicians at West China Hospital used the telemedicine platform
Ma[23]	Hotlines	Psychological assistance	Rehabilitation (physiotherapy, speech therapy, occupational therapy) via prescribe exercise videos and reminders to patients	Cellphones	/	Utilization: number of calls received at the regional level hotline (n = 3,206) was 0.021% of the population of the region.
Lai[24]	SMS	Ophthalmic outpatient clinic	Content of the SMS included (1) option of rebooking and drug refill via telephone hotline and (2) persuasion of patients with fever, flu symptoms or recent travel history to avoid ophthalmic clinic attendance	Cell phones	/	Effectiveness: Reduction of 13.9% of clinic attendance
Zhou[25]	Mobile application Good Doctor and Ali Medical Valley Online Platform	Multiple	Tele-consultation	Mobile application, internet connection	/	Utilization: Increased utilization during COVID (1,324 patients to 2,150 patients)
Zhang[26]	synchronous teleprogramming software	movement disorders	Deep brain stimulation, video communication, DBS telemedicine parameter adjustment	Computer, camera, wi-fi, internet connection	/	Utilization: 909 telemedicine sessions requested in total; Satisfaction: 89% sessions were perceived as satisfactory and clinically useful
Chen[27]	a teleophthalmology platform that used the videoconference platform for teleconsultation, after which telelaser planning and intervention were conducted with a laser system and a platform for remote computer control, which were connected via 5G networks.	diabetic retinopathy	telelaser planning and intervention, video conference	5G network, a laser system, computer, camera, remote control	Experience of visual acuity: 4 participants (7 eyes) did not experience	System performance: data upload and download speed, network latency
Li[28]	Drug supply management system, contactless drug	General	Medication and equipment ordering, electronic prescription and delivery, patient consultation with pharmacists, health education	Mobile phone, hospital information system	/	Utilization: 30,000 inquiries to clinical pharmacists

	delivery system using robots, cloud pharmacy system, wechat					
Lee[29]	A cross-platform real-time mobile application	/	Self-monitoring and management during home quarantine	Mobile phone, Bluetooth, personal temperature monitoring patch	/	Utilization: 2,088 users
Lin[30]	Remote management system	Psychiatric disorders	DBS teleprogramming, individualized psychological support, and medical consultations	Wifi, Bluetooth, computer	Assessment of OCD symptoms, comorbid symptoms of depression (BDI) and anxiety (BAI)	/
Ding[31]	Mobile phone-based miniprogram	Multiple	Remote consultation, online clinic appointment, video consultation, online prescription	Mobile phone, internet connection, cameras	/	Utilization: 1,380 patients, increased number of prescriptions after Week 5
Mu[32]	Mobile application (Haodaifu)	dermatology	Remote consultation	Internet connection, mobile phone, cameras and videos	/	Utilization: 698 patients used the system
Shao[33]	online medical APP, telemedicine, WeChat service, and consultation hotline, public education	Department of Respiratory Medicine, Infectious Diseases, Emergency Medicine, Critical Medicine	the complete pre- hospital screening and early detection and treatment.. Internet medical call center experts who are responsible for the hotline and provide 7 24 free services to the public. Patients consult online through text, images, video screens, and more. The hospital also organizes live online lectures by experts to promote popular scientific knowledge and healthcare skills to the public.	Mobile phone, internet connection, camera, video	/	Utilization: 84,255 consultations through the system; 15,510 follow-ups
Wang[34]	A remote consultation platform, Beijing ICF V5.1 System	Department of Telemedicine Consultation Center	Remote consultation, video and audio conferencing; remote imaging diagnosis and remote education	Ordinary computers and internet connection for "soft video"; Polycom audio and video systems for hard video	/	Utilization (patients): 1,043 consultations; Efficiency: 76.03% of the cases were finished in less than 2 hours; Utilization (medical professionals): 15,200 medical personnel from 666 hospitals used the remote education; Satisfaction (providers): 91.8%; Satisfaction (patients): 68.42%;
Han[35]	Mobile application	Chronic pain	Patients: Choosing a provider, making appointments for remote programming services, access medical history, and report daily outcomes. Providers: Storing patients' medical records, images, previous programming records	Internet connection, Bluetooth, implanted pulse generator, remote control	/	Utilization: 81.3% patients used the app more than twice; Satisfaction: 96.7%; Perceived clinical improvement: 96.7%; System failure: 4.8% patients
Zhang[36]	vital sign telemetry system; This system belongs to non-implanted noninvasive telemetry system consisting of ring-type medical pulse oximeter, non-contact radar respiratory monitor and remote vital signs telemetry platform	COVID-19	Collect and monitor vital signs such as blood oxygen saturation and blood pressure	Internet connection, sensor	/	Efficiency: the daily time per capita of the wards using the telemetry system is shorter than those without.

References

1. Ku, B.P.S., et al., *Tele-Rehabilitation to Combat Rehabilitation Service Disruption During COVID-19 in Hong Kong: Observational Study.* JMIR Rehabilitation and Assistive Technologies, 2021. **8**(3): p. e19946.
2. He, Q., et al., *Practice in Information Technology Support for Fangcang Shelter Hospital during COVID-19 Epidemic in Wuhan, China.* Journal of Medical Systems, 2021. **45**(4): p. 1-8.
3. Guo, Z., et al., *The safety and feasibility of the screening for retinopathy of prematurity assisted by telemedicine network during COVID-19 pandemic in Wuhan, China.* BMC ophthalmology, 2021. **21**(1): p. 1-8.
4. Fu, Z., et al., *Minimizing the Impact of the COVID-19 Epidemic on Oncology Clinical Trials: Retrospective Study of Beijing Cancer Hospital.* Journal of Medical Internet Research, 2021. **23**(3).
5. Lian, W., et al., *Digital health technologies respond to the COVID-19 pandemic in a tertiary hospital in China: development and usability study.* Journal of medical Internet research, 2020. **22**(11): p. e24505.
6. Wei, X., et al., *Evaluation on application of intelligent voice call system in popularizing knowledge of prevention and control of COVID-19 for chronic disease patients in the community.* Chinese Journal of General Practitioners, 2020: p. 388-393.
7. Lu, Y., et al., *Management of intractable pain in patients with implanted spinal cord stimulation devices during the COVID-19 pandemic using a remote and wireless programming system.* Frontiers in neuroscience, 2020. **14**: p. 1283.
8. Lee, A.K., et al., *Mitigation of head and neck cancer service disruption during COVID‐19 in Hong Kong through telehealth and multi‐institutional collaboration.* Head & Neck, 2020. **42**(7): p. 1454-1459.
9. Chen, M., et al., *Characteristics of online medical care consultation for pregnant women during the COVID-19 outbreak: cross-sectional study.* BMJ open, 2020. **10**(11): p. e043461.
10. Zhai, Y., et al., *An internet-based multidisciplinary online medical consultation system to help cope with pediatric medical needs during the COVID-19 outbreak: a cross-sectional study.* Translational Pediatrics, 2021. **10**(3): p. 560.
11. Liu, G., et al., *The efficacy of WeChat-Based parenting training on the psychological well-being of mothers with children with autism during the COVID-19 Pandemic: Quasi-experimental study.* JMIR Mental Health, 2021. **8**(2): p. e23917.
12. Zhang, Q.-L., et al., *Telemedicine usage via WeChat for children with congenital heart disease preoperatively during COVID-19 pandemic: a retrospective analysis.* International Journal for Quality in Health Care, 2021. **33**(2): p. mzab066.
13. Zhang, S., et al., *Changes in home blood pressure monitored among elderly patients with hypertension during the covid-19 outbreak: a longitudinal study in China leveraging a smartphone-based application.* Circulation: Cardiovascular Quality and Outcomes, 2021. **14**(5): p. e007098.
14. Wong, A.K.C., et al., *Effect of a telecare case management program for older adults Who Are homebound during the COVID-19 pandemic: a pilot randomized clinical trial.* JAMA network open, 2021. **4**(9): p. e2123453-e2123453.
15. Xu, H., et al., *Monitoring and management of home-quarantined patients with COVID-19 using a WeChat-based telemedicine system: retrospective cohort study.* Journal of medical Internet research, 2020. **22**(7): p. e19514.
16. Li, L., et al., *Management of follow-up with preterm infants during the outbreak in China.* Frontiers in Pediatrics, 2021. **9**: p. 387.
17. Nan, J., et al., *Comparison of clinical outcomes in patients with ST elevation myocardial infarction with percutaneous coronary intervention and the use of a telemedicine app before and after the COVID-19 pandemic at a center in Beijing, China, from August 2019 to March 2020.* Medical Science Monitor: International Medical Journal of Experimental and Clinical Research, 2020. **26**: p. e927061-1.
18. Liu, L., et al., *Application and preliminary outcomes of remote diagnosis and treatment during the COVID-19 outbreak: retrospective cohort study.* JMIR mHealth and uHealth, 2020. **8**(7): p. e19417.
19. Li, H.-L., et al., *Pilot study using telemedicine video consultation for vascular patients' care during the COVID-19 period.* Annals of vascular surgery, 2020. **68**: p. 76-82.
20. Li, H., et al., *The Establishment and Practice of Pharmacy Care Service Based on Internet Social Media: Telemedicine in Response to the COVID-19 Pandemic.* Frontiers in Pharmacology, 2021. **12**.
21. Xia, W., et al., *A telerehabilitation programme in post-discharge COVID-19 patients (TERECO): a randomised controlled trial.* Thorax, 2021.
22. Li, P., et al., *How telemedicine integrated into China's anti-COVID-19 strategies: case from a National Referral Center.* BMJ health & care informatics, 2020. **27**(3).
23. Ma, R., R. Nguyen, and J.M. Oakman, *Dissemination strategies and usage of psychological assistance hotlines during the COVID-19 outbreak in China.* Frontiers in Communication, 2020: p. 60.
24. Lai, T.H., et al., *The use of short message service (SMS) to reduce outpatient attendance in ophthalmic clinics during the coronavirus pandemic.* International ophthalmology, 2021. **41**(2): p. 613-620.

25. Zhou, F., et al., *Online clinical consultation as a utility tool for managing medical crisis during a pandemic: retrospective analysis on the characteristics of online clinical consultations during the COVID-19 pandemic.* Journal of Primary Care & Community Health, 2020. **11**: p. 2150132720975517.
26. Zhang, C., et al., *Utility of deep brain stimulation telemedicine for patients with movement disorders during the COVID-19 outbreak in China.* Neuromodulation: Technology at the Neural Interface, 2021. **24**(2): p. 337-342.
27. Chen, H., et al., *Application of 5G Technology to Conduct Real-Time Teleretinal Laser Photocoagulation for the Treatment of Diabetic Retinopathy.* JAMA ophthalmology, 2021. **139**(9): p. 975-982.
28. Li, Z., et al., *Innovative Strategies and Efforts of Clinical Pharmacy Services During and After COVID-19 Epidemic: Experience from Shanghai Children's Hospital.* Risk Management and Healthcare Policy, 2021. **14**: p. 4759.
29. Lee, S.H., et al. *Establish a Real-time Responsible Home Quarantine and Monitoring Management mHealth Platform*. in *AMIA Annual Symposium Proceedings*. 2020. American Medical Informatics Association.
30. Lin, Z., et al., *Deep brain stimulation telemedicine programming during the COVID-19 pandemic: treatment of patients with psychiatric disorders.* Neurosurgical focus, 2020. **49**(6): p. E11.
31. Ding, L., et al., *The internet hospital plus drug delivery platform for health management during the COVID-19 pandemic: observational study.* Journal of Medical Internet Research, 2020. **22**(8): p. e19678.
32. Mu, Z., et al., *Teledermatology Service During the COVID-19 Pandemic in China: A Mobile Application-Based Retrospective Study.* Clinical, cosmetic and investigational dermatology, 2021. **14**: p. 1119.
33. Shao, C., et al., *Taizhou's COVID-19 prevention and control experience with telemedicine features.* Frontiers of medicine, 2020. **14**(4): p. 506-510.
34. Wang, Y., et al., *Application of telemedicine in the COVID-19 epidemic: An analysis of Gansu Province in China.* Plos one, 2021. **16**(8): p. e0249872.
35. Han, Y., et al., *The use of remote programming for spinal cord stimulation for patients with chronic pain during the COVID-19 outbreak in China.* Neuromodulation: Technology at the Neural Interface, 2021. **24**(3): p. 441-447.
36. Zhang, J., et al., *Application value of vital signs telemetry system for 2019 novel coronavirus disease suspected cases in isolation wards.* Infection and Drug Resistance, 2020. **13**: p. 2971.
